# Plant Surface Cues Prime *Ustilago maydis* for Biotrophic Development

**DOI:** 10.1371/journal.ppat.1004272

**Published:** 2014-07-17

**Authors:** Daniel Lanver, Patrick Berndt, Marie Tollot, Vikram Naik, Miroslav Vranes, Tobias Warmann, Karin Münch, Nicole Rössel, Regine Kahmann

**Affiliations:** 1 Max Planck Institute for Terrestrial Microbiology, Department of Organismic Interactions, Marburg, Germany; 2 Karlsruhe Institute of Technology (KIT), Institute for Applied Biosciences, Department of Genetics, Karlsruhe, Germany; Temasek Life Sciences Laboratory, Singapore

## Abstract

Infection-related development of phytopathogenic fungi is initiated by sensing and responding to plant surface cues. This response can result in the formation of specialized infection structures, so-called appressoria. To unravel the program inducing filaments and appressoria in the biotrophic smut fungus *Ustilago maydis*, we exposed cells to a hydrophobic surface and the cutin monomer 16-hydroxy hexadecanoic acid. Genome-wide transcriptional profiling at the pre-penetration stage documented dramatic transcriptional changes in almost 20% of the genes. Comparisons with the *U. maydis sho1 msb2* double mutant, lacking two putative sensors for plant surface cues, revealed that these plasma membrane receptors regulate a small subset of the surface cue-induced genes comprising mainly secreted proteins including potential plant cell wall degrading enzymes. Targeted gene deletion analysis ascribed a role to up-regulated GH51 and GH62 arabinofuranosidases during plant penetration. Among the *sho1*/*msb2*-dependently expressed genes were several secreted effectors that are essential for virulence. Our data also demonstrate specific effects on two transcription factors that redirect the transcriptional regulatory network towards appressorium formation and plant penetration. This shows that plant surface cues prime *U. maydis* for biotrophic development.

## Author Summary

A basic requirement for pathogens to infect their hosts and to cause disease is to detect that they are in contact with the host surface. Plant pathogenic fungi typically respond to leaf surface contact with the development of specialized infection structures enabling the fungus to penetrate the leaf cuticle and to enter the plant tissue. In this study we analyzed the response of the corn smut fungus *Ustilago maydis* to two plant surface cues, such as hydrophobic surface and cutin monomers. Based on genome-wide gene expression analysis we found that these cues trigger the production of secreted plant cell wall degrading enzymes helping the fungus to penetrate the plant surface. In addition, genes were activated that code for a group of secreted proteins, so-called effectors, that affect virulence after penetration. These results demonstrate that plant surface cues trigger fungal penetration of the plant surface and also prime the fungus for later development inside plant tissue. These specific responses required two cell surface proteins that likely function as plant surface sensors.

## Introduction

Plant pathogenic fungi have developed sophisticated strategies to attach to and subsequently invade their host, often by undergoing distinct morphological changes during this part of the life cycle. These include filament formation upon recognition of the host surface and development of specialized infection structures. Such structures, appressoria or hyphopodia, facilitate penetration of the host surface either by localized secretion of lytic enzymes or mechanical force. The host surface provides a variety of physical and chemical stimuli that are perceived by fungi and that induce differentiation and appressorium formation [1]–[3]. Appressorium formation is a complex, highly regulated process involving cell wall modification [4]–[8] changes in gene regulation [9]–[14] and in cytoskeletal organization [15]–[18]. Learning about the molecular mechanisms that are required for perception and integration of these stimuli is crucial to understand how plant pathogens enter their host.

The *Ustilago maydis* – *Zea mays* pathosystem has emerged as the current model for plant pathogenic basidiomycetes and as one of the few models for a true biotrophic interaction that persists throughout fungal development inside the host plant [19]–[21]. For colonization of its host plant maize, *U. maydis* is equipped with a large set of novel secreted effectors which are needed to suppress plant defense responses and to reprogram the host metabolism [22]. Pathogenic development of *U. maydis* is initiated by the filamentous dikaryon that is generated after pheromone-induced fusion of two compatible haploid *U. maydis* cells. Filamentation requires the heterodimeric bE/bW transcription factor [19]. The dikaryotic filament grows unipolar, and retraction septa delimit the cytoplasm-filled tip compartment from the older parts of the filament [23]. On the plant surface, the tip cell eventually differentiates an appressorium, which facilitates penetration of the plant cuticle and cell wall. In contrast to the dome-shaped melanized appressoria of *Magnaporthe oryzae* or *Colletotrichum spp.* that penetrate mainly by high turgor pressure [24], [25], appressoria of *U. maydis* are non-melanized and are proposed to penetrate the cuticle by secretion of plant cell wall degrading enzymes (CWDEs) [26]. Knowledge on how non-melanized appressoria penetrate the plant surface is sparse and comes from studies on the maize pathogen *Cochliobolus carbonum*, where many of the CWDE genes are subject to catabolite repression [27]. After deletion of *snf1*, the activator of catabolite-repressed genes, a strong reduction in virulence is observed in *C. carbonum*, which could be attributed to reduced penetration efficiency [27]. In *U. maydis* catabolite repression is largely independent from *snf1* and deletion of *snf1* did not profoundly affect virulence [28].

In fungal pathogens, appressorium formation is regulated by a conserved MAP kinase cascade [29]. In *U. maydis*, this MAP kinase cascade signals in both, pheromone response and appressorium formation. The pheromone signal is perceived by a pheromone receptor and is further transmitted via the MAPKKK Kpp4, the MAPKK Fuz7 and the MAPK Kpp2 [30]. Kpp2 phosphorylates the pheromone response factor Prf1, which regulates the expression of the *a* and *b* mating type genes, encoding the pheromone/pheromone receptor system and the heterodimeric transcription factor, respectively. After cell fusion, the active bE/bW heterodimer triggers the hierarchical expression of downstream transcription factors that together regulate 345 genes [31]. More than 90% of the *b*-regulated genes are regulated by Rbf1, a direct bE/bW target [31]. Rbf1 induces among others the expression of the transcription factors *hdp1* and *biz1* that are involved in filament formation and virulence, respectively [31]–[33]. While pheromone signaling alone can induce cell fusion and filamentous growth, it does not induce appressorium formation. The major stimulus for inducing appressorium formation in *U. maydis* is a hard, hydrophobic surface [34]. In addition, chemical signals such as the cutin monomer 16-hydroxy hexadecanoid acid strongly enhance appressorium formation efficiency [34]. While the MAP-kinase Kpp2 is crucial for filament and appressorium formation on a hydrophobic surface [34], a second MAP-kinase, Kpp6, is needed for appressorium function, i.e. penetration of the plant surface [35]. Furthermore, two plasma membrane proteins, Sho1 and Msb2, that act upstream of the MAP-kinases Kpp2 and Kpp6, specifically regulate appressorium formation in response to the hydrophobic surface [36], [37]. Sho1 and Msb2 are conserved proteins in fungi that regulate host penetration via MAP kinase signaling also in the phytopathogenic fungi *Fusarium oxysporum* and *M. oryzae* [38], [39].

A thorough analysis of the transcriptome at the stage of appressorium formation in phytopathogenic fungi has been carried out for the ascomycete fungi *M. oryzae* and *Botrytis cinerea*, a hemibiotrophic pathogen of rice and a necrotrophic pathogen with a broad host range, respectively [11], [12], [14]. In this study, we investigated the transcriptional changes during early pathogenic development of the biotroph *U. maydis*. We used the previously established *in vitro* system for infection-related development [34], which allowed us to monitor transcriptional changes at the pre-penetration stage, i.e. during filamentation and appressorium formation. We could show that sensing of plant surface cues induces the expression of genes encoding plant cell wall degrading enzymes that contribute to the ability of *U. maydis* to penetrate the plant surface. On the other hand, plant surface cues induced expression of known secreted effectors that are needed specifically for biotrophic development after penetration. These responses were dependent on the putative sensors for hydrophobic surface Sho1 and Msb2. Thus, perception of plant surface cues primes *U. maydis* for biotrophic development.

## Results/Discussion

### Hydrophobic surface and hydroxy fatty acids induce major changes in the transcriptome of *U. maydis*

Transcriptional profiling during early pathogenic development of *U. maydis* was performed using custom Affymetrix arrays (MPIUstilagoA) covering 5823 of the predicted 6849 *U. maydis* genes. To induce appressoria the solopathogenic AM1 strain was used. This solopathogenic strain does not require a mating partner for inducing filaments and appressoria, and carries an appressorium specific GFP-reporter construct [34]. The transcriptional response of *U. maydis* to a hydrophobic surface (HS) was monitored, by spraying AM1 in low nutrient medium on ParafilmM and incubating for 12 h (see Materials and Methods). This time point was chosen as the transition from yeast-like cells to filaments had occurred efficiently and about 5% of the filaments had started to develop appressoria. This number did not increase upon prolonged incubation but resulted in re-growth of hyphae from appressoria (not shown), i.e. the 12 h time point represents the pre-penetration time point. To investigate the transcriptional response to the combination of hydrophobic surface and hydroxy fatty acids (HS+FA), the AM1 culture was supplemented with 100 µM 16-hydroxy hexadecanoid acid (HDA) prior to spray-inoculation of ParafilmM. After 12 h incubation 20% of the filaments had started to develop appressoria. To create a reference data set for the experiment, cells of the AM1 strain were sprayed on a hydrophilic glass surface and incubated for 2 h (glass control, GC). This time point was chosen to allow cell sedimentation and the adaption to the low nutrient medium. In addition, this time point allowed to avoid nutrient depletion stress which would have occurred if undifferentiated cells had been incubated for 12 h, the time needed to observe differentiated cells on the hydrophobic surface. The comparison of three biological replicates generated under the three conditions (HS, HS+FA, GC) allowed us to monitor the transcriptional response of *U. maydis* to the physical and chemical plant-derived surface cues.

**Figure 1:**
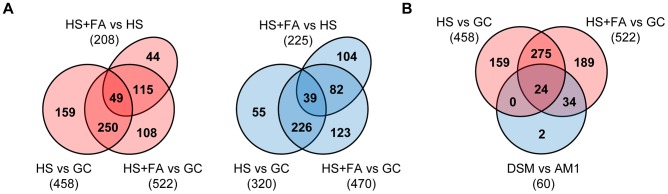
Venn diagram illustrating overlaps between numbers of genes that are differentially regulated in response to plant surface-cues. A. Overlaps of up-regulated (red) and down-regulated (blue) genes during differentiation from budding cells to filaments (HS vs GC), budding cells to appressoria (HS+FA vs GC) and filaments to appressoria (HS+FA vs HS). B. Overlaps of up-regulated genes (red) during differentiation from budding cells to filaments and appressoria (HS vs GC and HS+FA vs GC, respectively) and down-regulated genes (blue) in AM1Δsho1Δmsb2 (DSM) compared to AM1 (DSM vs AM1), both incubated on HS+FA. HS: Hydrophobic surface, FA: Fatty acid, GC: Glass control.

We compared the transcriptional changes between filaments and sporidia (HS vs GC), between appressoria and yeast-like cells (HS+FA vs GC), and between appressoria and filaments (HS+FA vs HS). Genes that showed a fold-change ≥2 (p-value ≤0.05 with FDR of 0.01) were considered to be differentially expressed. By this analysis, 725 genes were found to be induced either by the hydrophobic surface or by the combination of hydrophobic surface and fatty acid signal, and 629 genes were found to be repressed by these cues (Figure 1A and Table S1). For 6 of the surface-cue induced genes the microarray data was confirmed by quantitative real-time PCR (Figure S1). Based on the expression pattern, we categorized the identified 1223 differentially regulated genes in 8 different groups: 1) HS- and FA-induced (170 genes); 2) HS-induced (264 genes); 3) FA-induced (92 genes); 4) HS-induced, FA-repressed (166 genes); 5) HS-repressed, FA-induced (71 genes); 6) FA-repressed (69 genes); 7) HS-repressed (246 genes); 8) HS- and FA-repressed (145 genes; Table S1). Thus, the plant surface cues induce major changes in the transcriptome of *U. maydis*, reflecting the switch from saprophytic growth to pathogenic development. Of the 526 HS and FA induced genes (group 1, 2 and 3) 137 have been previously identified to be up-regulated in tumors of infected maize plants when compared to *U. maydis* grown in axenic culture [21]. Only 62 of the HS and FA-induced genes were found repressed in tumors. In accordance with that, 173 of the 460 HS and FA-repressed genes (group 6, 7 and 8) are also repressed in tumors while only 51 of the HS and/or FA-repressed genes are induced in tumor tissue [21]. This correlation suggests that sensing and responding to plant surface cues induces an adaption that is to a significant extent maintained during subsequent biotrophic development.

### The plasma membrane proteins Sho1 and Msb2 are responsible for the expression of a subset of the surface cue-induced genes

We were particularly interested in genes that are specifically expressed at the appressorial stage. Therefore we included in our microarray experiment the AM1Δsho1Δmsb2 strain, a mutant that is unable to form appressoria in response to the hydrophobic surface [36]. Only 6 genes were found to be differentially regulated in the mutant compared to AM1 when grown on the hydrophobic surface without addition of hydroxy fatty acids (Table S2). Under efficient appressorium-inducing conditions (HS+FA), 61 differentially regulated genes were identified (Table S2). Of these genes 60 were down-regulated in the Δ*sho1*Δ*msb2* mutant, including the 6 genes that were identified as *sho1/msb2* dependently expressed on the hydrophobic surface alone. Interestingly, 58 of these 60 genes belong to the HS and/or FA-induced genes (Figure 1B and Table S2), demonstrating that Sho1 and Msb2 are necessary for the induction of a subset of the surface-cue induced genes. Given that on the hydrophobic surface only 5% of the filaments develop appressoria, the minor difference between the transcriptome of the Δ*sho1*Δ*msb2* mutant compared to the AM1 strain under this condition is likely due to those few AM1 hyphae that have differentiated an appressorium. Since the difference in the transcriptome of the Δ*sho1*Δ*msb2* mutant and the AM1 strain was much more evident under efficient appressorium inducing conditions (HS+FA), this suggests that the *sho1 msb2* double mutant arrests at a stage before appressorium formation.

### Plant surface cues induce the expression of secreted effector proteins needed for biotrophic development

**Figure 2:**
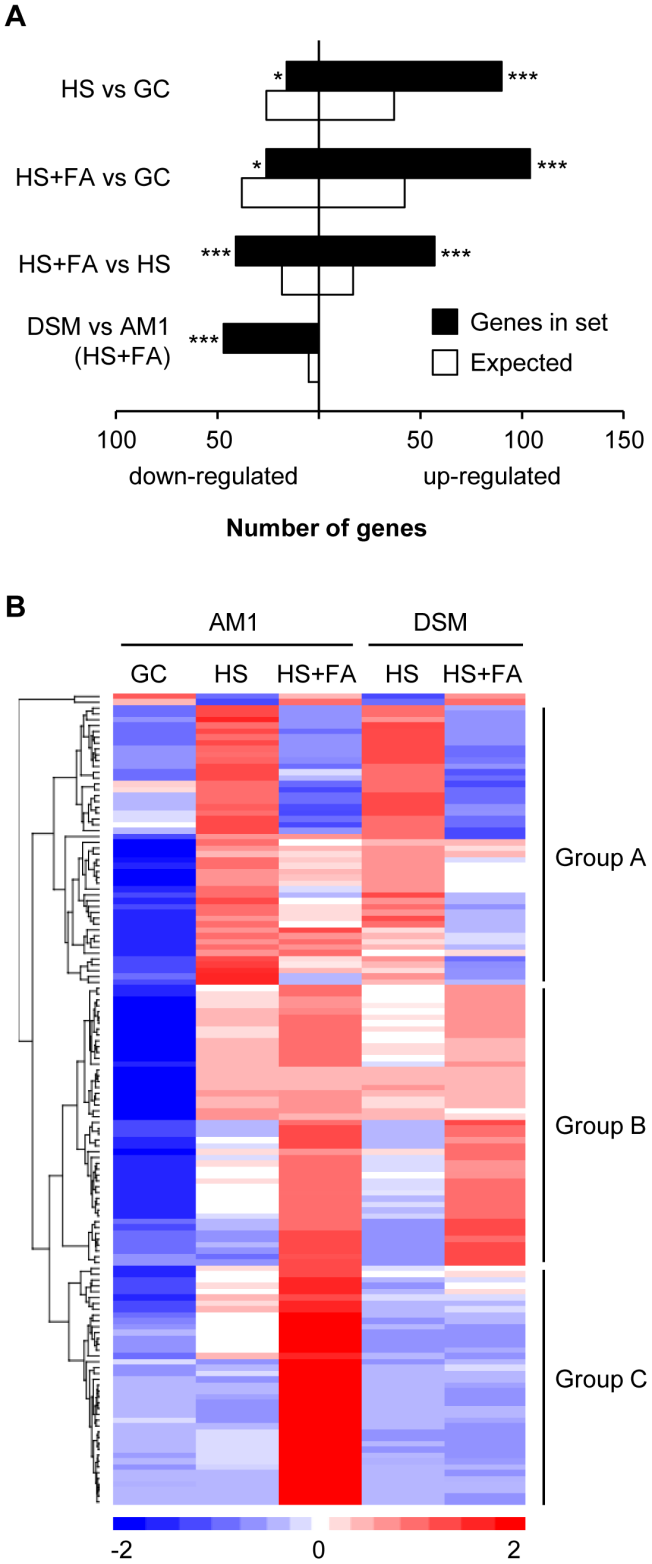
Genes encoding secreted proteins are transcriptionally induced by hydrophobicity and hydroxy fatty acids. **A**. Expression of genes encoding putative secreted proteins is significantly enriched during appressorium formation. For each gene set the number of genes encoding proteins predicted to be secreted is compared to the expected number of genes predicted from the whole genome sequence. *, ** and *** denote p-values (hypergeometric distribution) of p<0.01, p<0.001 and p<0.0001, respectively. **B**. The heat-map depicts the expression of genes encoding putative secreted proteins that were found to be up-regulated in at least one of the following comparisons: HS vs GC, HS+FA vs HS and HS+FA vs GC. Expression is visualized for the AM1 strain (GC, HS, HS+FA) and the AM11Δsho1Δmsb2 (DSM) strain (HS, HS+FA). The order of genes was defined by hierarchical clustering [42] resulting in the classification of three major groups: Group A: HS-induced and predominantly FA-repressed. Group B: HS and FA-induced. Group C: HS and/or FA-induced, *sho1*/*msb2* dependent. All genes are listed in Table S3. HS: Hydrophobic surface, FA: Fatty acid, GC: Glass control, DSM: AM1Δsho1Δmsb2.

To establish compatibility, biotrophic pathogens need to overcome the PAMP (pathogen-associated molecular pattern)-triggered plant defense mechanisms [40]. To achieve this, microbial pathogens secrete so-called effectors, various secreted proteins, that interfere with the plant immune response and facilitates compatibility [40]. A hypergeometric enrichment test revealed that during filament- and appressorium formation genes encoding secreted proteins were significantly over-represented among the induced genes and under-represented among the repressed genes (Figure 2A). Of the 554 *U. maydis* genes predicted to encode secreted proteins [41], 139 genes were induced during filament- and appressorium formation. Remarkably, of the 60 genes that require *sho1* and *msb2* for their induction 46 encode proteins predicted to be secreted (Figure 2 and Table S2). This indicates a link between plant surface cue perception and expression of secreted proteins. By hierarchical clustering [42] the 139 surface cue-induced genes predicted to encode secreted proteins were categorized into three major groups (Figure 2B and Table S3). Group A includes 47 genes that were HS-induced and predominantly FA-repressed. Group B includes 48 genes that were HS and FA-induced. In group C, which comprises 42 genes, genes were *sho1*/*msb2*-dependently induced by HS and/or FA. In this latter group we found important virulence factors like *cmu1* (*um05731*), *pit2* (*um01375*) and *stp1* (*um02475*). Cmu1 is a secreted chorismate mutase that is taken up by plant cells where it suppresses salicylic acid synthesis [43]. Pit2 is a secreted effector with protease inhibitor activity that is proposed to function in conjunction with the transmembrane protein Pit1 to maintain biotrophy during late stages of plant infection [44], [45]. Interestingly, *pit1* shows a similar expression profile as *pit2*, i.e. is induced by the surface cues and requires *sho1* and *msb2* for induction (Table S2). *stp1* encodes a secreted effector that is essential for the initial establishment of hyphae in the epidermal cell layer [46]. All these effectors have in common that they have their specific function after penetration of the plant surface and are not involved in saprophytic growth or in the penetration process itself [43], [44], [46]. Many *U. maydis* effectors are organized in gene clusters [21], [47], e.g. *stp1* resides in cluster 5B. Besides *stp1*, group C contained all three genes of cluster 8A (*um03201, um03202, um10403*), 4 of 6 cluster 2B genes (*um01297, um01299, um01300, um01301*), two genes (*um06180, um06181*) of the *mig2* cluster and the paralog mig2-6 (*um06126*) located elsewhere in the genome [48], two genes of cluster 12-15 (*um10418, um12258*), as well as single genes of cluster 11-16 (*um11062*), 17-15 (*um04816*) and 1-32 (*um00792*). Although for these genes a virulence function has not yet been demonstrated, it is tempting to speculate that many of the surface-cue induced genes predicted to encode secreted proteins function as effectors and contribute to the establishment of biotrophy. While most of the effectors were present in group C, another characterized effector, *pep1* (*um01987*) was found in group A of the HS-induced, FA-repressed genes. Pep1 is needed to suppress the oxidative burst during penetration of the plant [49]. *pep1* expression is induced during colonization and stays on during all stages of biotrophic development [50]. The fact that we detect a down-regulation by FA suggests that additional factors present on the plant surface or during penetration but absent in the *in vitro* system contribute to the expression of this gene. Little is known about the signals that trigger effector gene expression in eukaryotic plant pathogens. Nitrogen limitation can induce the expression of some fungal effectors *in vitro* though it is unlikely that nitrogen limitation is the major trigger inside plant tissue [51]. Our results clearly show that expression of secreted effectors is a key response to sensing plant surface cues. With Sho1 and Msb2 being the receptors for perceiving the surface signals we could identify for the first time membrane-bound receptors that trigger effector gene expression in a plant pathogen. During appressorium formation of *M. oryzae* genes predicted to encode secreted proteins were also found to be enriched among the induced genes [11]. However, it remains to be shown whether any of these effectors are required for the hemibiotrophic lifestyle of *M. oryzae*. In *B. cinerea* the cerato-platanin family protein SPL1 is transcriptionally induced during appressorium formation on apple wax surfaces [14]. This protein contributes to the necrotrophic lifestyle of the fungus by inducing a hypersensitive response in plant cells [52]. Overall, the results suggest that fungal plant pathogens become primed for their specific mode of development already prior to penetration when they are growing on the plant surface.

### Plant surface cues induce the expression of plant cell wall-degrading enzymes

For phytopathogenic fungi the first obstacle to overcome is the plant cuticle and the plant cell wall. It is generally assumed that non-melanized appressoria penetrate the plant surface through local enzymatic digestion of the plant cell wall. However, in *U. maydis*, attempts to identify CWDEs with a virulence function have not yet been successful, most likely due to gene redundancy. We expected that during appressorium formation genes encoding potential CWDEs are transcriptionally induced. Of 81 secreted carbohydrate active enzymes (CAZy) [53] encoded by the *U. maydis* genome, 38 are potentially involved in the modification of the plant cell wall (Table S4). Of those genes 15 were found to be differentially expressed during filament and appressorium formation and 7 of them belong to group C (*um11211*, *um12233*, *um04422*, *um02523*, *um04816*, *um00876*, *um01829*). *um11211* encodes a putative cutinase that could be involved in the initial digestion of the plant cuticle. *um12233* encodes a pectin methylesterase. However, pectin is only a minor component of the maize cell wall and pectinolytic enzymes of *U. maydis*, including *um12233* have been shown previously to be not involved in pathogenicity of *U. maydis* [54]. Two genes encoding GH128 β-1,3-glucanases (*um05528*, *um05704*) were highly induced during filament and appressorium formation. These enzymes could either modify the fungal cell wall, or degrade callose depositions [55].

The cell wall of maize epidermal cells consists mainly of cellulose (60%) and hemicellulose (30%) [56]. Strikingly, all three cellulases of the GH45 family in *U. maydis*, *um06332* (*egl1*), *um02523* (*egl2*) and *um04816* (*egl3*), were highly induced during filament and appressorium formation and the latter two belong to group C, i.e. were *sho1*/*msb2*-dependently expressed (Table S4). The endoglucanase *egl1* (*um06332*) has been identified previously as filament-specifically expressed gene but its deletion did not affect virulence [57]. To assess redundancy of GH45 enzymes, all three genes were co-deleted in the solopathogenic SG200 strain. Maize plants were infected with the triple mutant and the disease symptoms were scored according to severity as illustrated in Figure S2. However, in such infections SG200Δ3egl was as virulent as the SG200 strain (Figure S3). Likewise, appressorium and filament formation were not affected (Figure S3). The role of cellulases for plant pathogenic fungi is subject of controversial debate [58]. While the biotroph *Blumeria graminis* lacks canonical enzymes for cellulose degradation [59], the hemibiotroph *M. oryzae* has a large diversity of cellulases, and enzymes of the GH6 and GH7 family have been demonstrated to be involved in penetration and virulence [60]. In *U. maydis* the repertoire of cellulases is limited to GH45, GH3 and GH5 family members of which only the GH45 enzymes were up-regulated by the surface cues (Table S4). However, this does not exclude the possibility that GH5 and GH3 enzymes can substitute for GH45 enzymes during pathogenic development of *U. maydis*. Alternatively, cellulose degradation plays no role during plant colonization by *U. maydis*.

**Figure 3:**
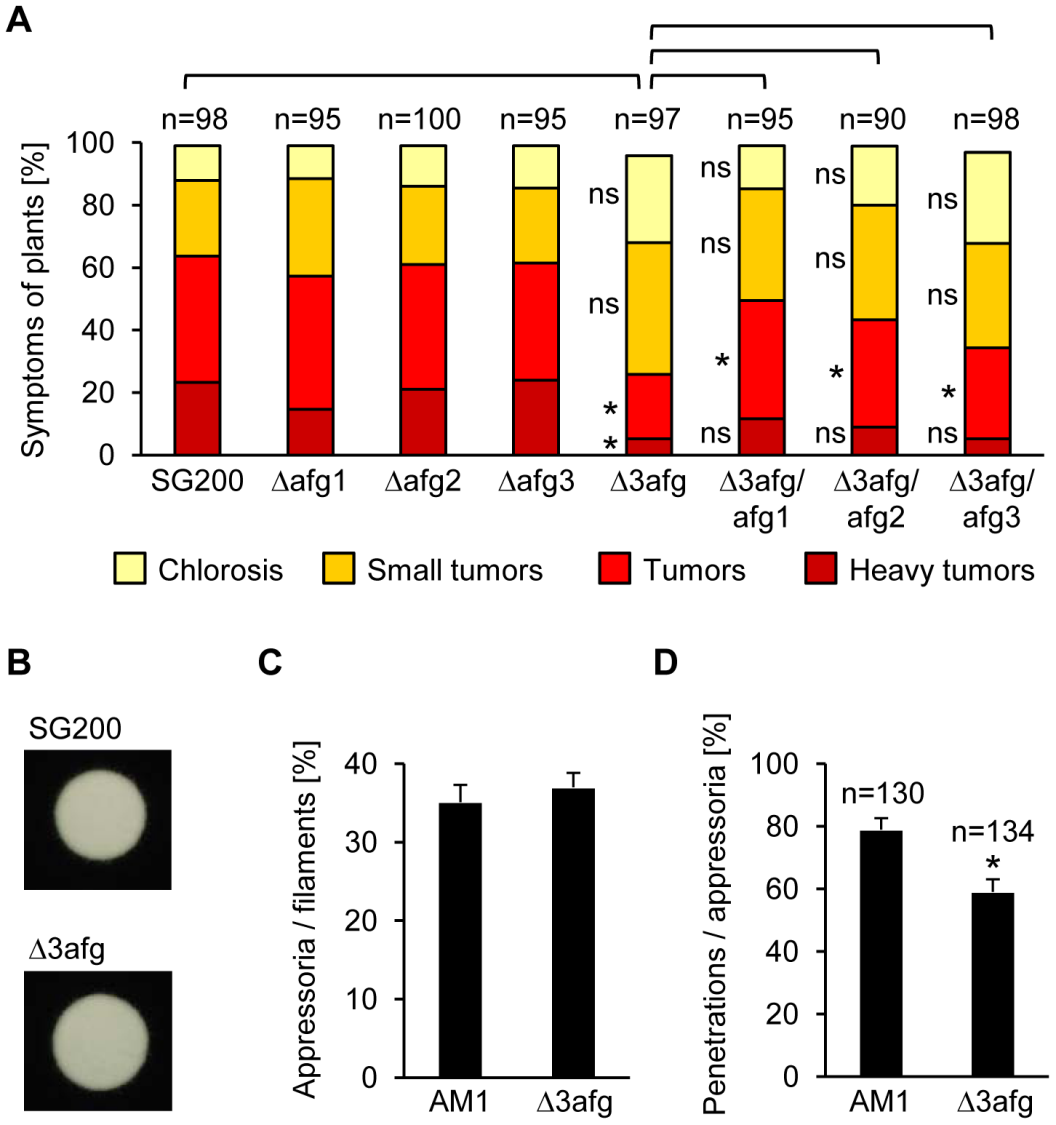
Arabinofuranosidases function in plant penetration and are needed for full virulence. **A**. Disease symptoms caused by SG200, *afg* single mutants and *afg* triple mutants as well as derivates of the latter strain, complemented with either *afg1*, *afg2* or *afg3*. The indicated strains were injected into maize seedlings and symptoms were scored 12 days after infection according to severity; the color code for each category is given below. Three independent experiments were carried out and the average values are expressed as a percentage of the total number of infected plants (n), which is given above each column. *Significant difference for each category and pair given above (p<0.05, student's t) ns: not significant. Tumor formation is significantly reduced in SG200Δ3afg and this defect can be partially complemented by introducing either *afg1*, *afg2* or *afg3*. **B**. Filament formation. SG200 and SG200Δ3afg were spotted on PD charcoal plates and incubated for 24 h at 28°C. The white fuzzy colonies reflect the formation of *b*-dependent filaments. **C**. Appressorium formation. AM1 and AM1Δ3afg were sprayed on ParafilmM with 100 µM HDA and incubated for 18 h at 28°C. Hyphae were stained with calcofluor and the average percentage of cells that expressed the AM1 marker was determined relative to the cells that had formed filaments. In three independent experiments more than 400 cells per strain were analyzed and error bars indicate standard error. **D**. Penetration efficiency. The indicated strains were injected into maize seedlings and 20 h after inoculation the number of appressoria that have penetrated the epidermis relative to the total number of appressoria (n) was determined. Three independent experiments were conducted and the error bars denote standard error. *Significant difference (p<0.05, student's t).

The main component of hemicellulose in maize cell walls is arabinoxylan [56]. Efficient degradation of arabinoxylan requires release of arabinofuranose side chains by α-L-arabinofuranosidases, as this makes the polymer more accessible to xylanases [61]. *U. maydis* has two GH51 α-L-arabinofuranosidases (*um01829, um00837*). The GH51 arabinofuranosidase *um01829* (*afg1*) was 12-fold induced during appressorium formation and required *sho1* and *msb2* for induction (Table S2 and S4). GH51 arabinofuranosidases have been shown to share their enzymatic activity against arabinoxylan with GH62 enzymes [62] and *U. maydis* encodes one member of GH62 (*um04309*). The arabinofuranosidase genes *um00837* (*afg2*) and *um04309* (*afg3*) were not represented on the Affymetrix array. Therefore, we performed quantitative real-time PCR which revealed that *afg3* is 4-fold induced during appressorium formation while *afg2* is not (Figure S4). Single deletions of *afg1*, *afg2* and *afg3* did not affect virulence but a triple deletion strain (SG200Δ3afg) caused significantly less severe symptoms (tumors and heavy tumors) on infected maize plants than the SG200 control strain (Figure 3A). We observed partial complementation when either *afg1*, *afg2* or *afg3* were re-integrated into the genome of SG200Δ3afg (Figure 3A). While filament and appressorium formation were not affected by the triple deletion (Figure 3B and C), appressoria of the mutant strain had a reduced penetration efficiency on maize plants (Figure 3D), which most likely explains the reduced virulence. In *Sclerotinia sclerotiorum* a GH54 arabinofuranosidase, Ssaxp, is required for full virulence on canola plants [63] and in *M. oryzae* a GH51 as well as a GH62 enzyme are induced during appressorium formation [12]. We consider it likely that also in those fungi arabinofuranosidases are involved in the plant penetration process.

Notably, some GH families were differentially expressed. For example one GH16 xyloglucanase (*um01898*) was induced during development on the hydrophobic surface while two GH16 members (*um04368, um05036*) were repressed. Likewise one GH10 β-1,4-endoglucanase (*um04422*) was induced during appressorium formation and another one (*um03411*) was repressed at this stage (Table S4).

During infection, plant cell wall components can act as DAMPs (damage-associated molecular patterns) and trigger plant defense responses [64]. It is possible that specific CWDEs are down-regulated during appressorium formation to lower the potential to trigger plant defence responses, i.e. the release of elicitors produced by these enzymes. A differentially controlled expression of CWDEs might therefore be necessary to facilitate covert penetration of the plant surface.

In summary, our observation that hydrophobicity and cutin monomers induce the expression of genes encoding plant cell wall degrading enzymes like pectinases, cellulases and arabinofuranosidases demonstrates that the very first contact with the plant surface is already sufficient to prepare the fungus for penetration of the cell wall. Whether hyphal exposure to the respective substrates of these enzymes, i.e. pectin, cellulose and hemicellulose leads to an additional enhancement of expression during the penetration process is currently unknown.

### Changes in the fungal cell wall during *in vitro* differentiation of filaments and appressoria

Chitin synthases are crucial for hyphal growth, cell wall integrity and appressorium formation [65], [66]. During filamentation (HS vs GC) we identified 4 induced chitin synthases (CHS), *chs1* (*um10718*), *chs6* (*um10367*), *chs7* (*um05480*) and *mcs1* (*um03204*). The latter three are important for pathogenic development [67]–[69]. Interestingly, we also could identify a SKT5 like gene (*um10641*) as being up-regulated during filament formation (HS vs GC; Table S1). SKT5 acts as CHS activator in *S. cerevisiae* [70], consistent with strong chitin synthase activity during filamentous growth. During appressorium formation (HS+FA vs GC) no significant changes in expression of the CHSs could be detected compared with cells forming filaments. Even though chitin is an important structural component of the fungal cell wall, it also acts as elicitor for the plant defense system and is degraded by chitinases further enhancing elicitation [71]–[73]. Since chitosan, the N-deacetylated form of chitin, is a poor substrate for chitinases [74], conversion of chitin to chitosan may protect the fungal cell wall from degradation by extracellular plant chitinases [75]. This conversion is facilitated by chitin deacetylases (CDA) and synthesis of these enzymes correlates with a lack of surface exposed chitin [76]. Two CDAs were induced (*um11922, um01788*) during appressorium formation, one CDA was HS-induced and FA-repressed (*um01143*) and another one (*um00638*) was HS-repressed and FA-induced (Table S4). This suggests that during filament and appressorium formation chitin is converted to chitosan to mask the growing hyphae. Extensive modification of the fungal cell wall during the morphological transition from yeast-like cells to filaments and appressoria is further supported by induced expression of a chitin binding protein (*um00330*), a chitinase (*um05290*), an α-mannosidase (*um01957*) and two β-1,3-glucanases (*um05528*, *um05704*).

One conserved signalling cascade that regulates cell morphology and cell wall integrity in fungi is the MOR/RAM pathway with its central NDR kinase [77]. The central NDR kinase in *U. maydis* is Ukc1 (Um04956). Deletion of *ukc1* or other components of the MOR pathway cause hyperpolarized growth, pigmentation and loss of pathogenicity [78], [79]. In contrast with most ascomycete fungi that possess one central NDR kinase, the basidiomycete *U. maydis* possesses a second NDR kinase (*um02741*, designated *ukc2*), which shows 45% amino acid identity with Ukc1. While *ukc1* was not differentially regulated, *ukc2* was 4-fold induced during filament formation (Table S5). To investigate the function of *ukc2*, we deleted the gene in the solopathogenic AN1 strain. AN1 is identical to AM1 except that it carries the appressorial marker gene construct ectopically inserted in the genome to free the *ip* locus for integration of complementation constructs. The mutant cells had no morphological phenotype but compared to the progenitor strain they were reduced in filamentous growth, produced 50% less appressoria and caused 70% less tumors in infected maize plants (Figure S5A–C). The AN1Δukc2 strain was sensitive to the cell wall stressor congo red (Figure S5D), indicating that Ukc2 in *U. maydis* functions in cell wall integrity. Whether Ukc2 is associated with the known MOR pathway or part of a separate pathway needs to be investigated.

In *U. maydis*, repellent peptides have functionally replaced hydrophobins [80]. The well characterized gene *rep1* [80]–[82], coding for the precursors of the repellent peptides responsible for the attachment of *U. maydis* hyphae to surfaces, was found to be dramatically up-regulated during filamentous growth and appressorium formation. This indicates that growth on the hydrophobic surface stimulates the ability of *U. maydis* hyphae to attach to this surface.

### Differential regulation of metabolism during *in vitro* differentiation of filaments and appressoria

To understand the cellular processes during filament and appressorium formation, the MIPS Functional Catalogue Database (FunCatDB) was used for determining over-represented cellular functions [83]. This revealed that during filament formation (HS vs GC) and during appressorium formation (HS+FA vs GC) genes involved in metabolism like amino acid biosynthesis and degradation, lipid metabolism, carbon compound metabolism, vitamin metabolism and secondary metabolism were significantly over-represented among the repressed genes (Figure S6), indicating that specific metabolic activities are lowered during the morphological transition on the hydrophobic surface. By contrast, in *M. oryzae* many metabolic pathways are induced during appressorium formation, in particular genes for fatty acid β-oxidation are highly expressed at this stage [11], [12]. In this system, oxidation of fatty acids has been suggested to be major supplier of energy and acetyl-CoA during appressorium formation [12]. In *U. maydis* the β-oxidation pathway was down-regulated during filament formation and even more repressed during appressorium formation (Table S5). We observed instead that the two key enzymes of glycolysis, 6-phosphofructokinase (*um11409*) and pyruvate kinasae (*um00157*) were transcriptionally induced during filament and appressorium formation (HS vs GC and HS+FA vs GC) while the gluconeogenesis-driving fructose-1,6-bisphosphatase (*um02703*) was repressed under the same conditions (Table S5). In addition, pyruvate dehydrogenase kinase (*um05275*), the negative regulator of the pyruvate dehydrogenase complex, was transcriptionally repressed. This suggests that during appressorium formation of *U. maydis* generation of energy and acetyl-CoA are mainly derived from glycolysis. Similar observations have been made for the powdery mildew fungus *B. graminis*, where genes for glycolytic enzymes were induced during appressorium formation [9].

While genes for enzymes of the TCA cycle were moderately down-regulated during appressorium formation of *U. maydis*, we found that two enzymes of the GABA (γ-aminobutyrate)-shunt, i.e. glutamate decarboxylase (*gad1*, *um06063*) and GABA transaminase (*gatA*, *um01080*), were up-regulated (Table S5). The GABA-shunt bypasses two enzymatic steps of the TCA cycle, resulting in one less molecule of guanosine triphosphate (GTP) for each molecule of α-ketoglutarate traversing the shunt. In *S. cerevisiae* GAD and the downstream components of the GABA shunt are required for oxidative stress response [84]. We take this and the fact that *U. maydis gad1* is induced during oxidative stress [85] as indication that the observed up-regulation of *gad1* and *gatA* during appressorium formation might prime *U. maydis* against oxidative burst during penetration. However, deletion of *gad1* and *gatA* did not affect virulence (Figure S3), and growth of the mutants under oxidative stress was not altered (Figure S7). In addition, the mutants were able to grow on medium with glutamate or GABA as sole carbon source (not shown). Since the genome of *U. maydis* encodes in total three glutamate decarboxylases and two putative GABA transaminases (Table S5), gene redundancy is the likely cause for the lack of mutant phenotypes.

Under nitrogen starvation *U. maydis* produces and secretes large amounts of the biosurfactants ustilagic acid and mannosylerythritol lipids (MELs) [86], [87]. While ustilagic acid has antimicrobial activity, the role of MELs for *U. maydis* is unclear. MELs are secreted by many microorganisms and they are generally involved in the attachment and detachment to and from solid surfaces [88]. Interestingly, we found that the genes for two key enzymes of MEL biosynthesis, *emt1* and *mac1*, encoding a glycosyltransferase and acyltransferase, respectively [86], are specifically induced during appressorium formation (Table S1) while another gene of the MEL biosynthesis cluster, *mat1*, encoding an acetyltransferase was down-regulated during appressorium formation. This suggests that *U. maydis* secretes deacetylated MELs during appressorium formation. Whether this contributes to surface attachment at the site of penetration, needs to be investigated.

### Differential regulation of transport processes during *in vitro* differentiation of filaments and appressoria

Based on our finding that plant surface cues induce the expression of effectors genes that are needed inside the plant to establish biotrophy, we also expected to find genes that facilitate nutrient uptake during biotrophic growth. Two oligo peptide transporters of the OPT superfamily (*um11057, um04347*) were highly up-regulated during appressorium formation (Table S6) and remained induced during tumor formation [21]. In plants, members of this family function as transporters of small peptides, glutathione, and metal-chelates [89]. Besides peptide transporters, 8 amino acid transporters were up-regulated during appressorium formation and three were down-regulated (Table S6). Induction of amino acid and peptide transporters during development has also been described in other plant pathogenic fungi, e.g. *Uromyces fabae* [90], [91], *F. oxysporum* [92] and *M. oryzae* [11]. This indicates that amino acids and peptides contribute to nutrition of *U. maydis* during its biotrophic phase. Five sugar transporters were down-regulated during filament and appressorium formation and two (*um03034* and *srt1*) were up-regulated (Table S6). Of these latter ones Srt1, a high affinity sucrose transporter, has been shown to be an important virulence factor needed for biotrophic growth [93]. By relying on the import of sucrose for biotrophic growth, the plant immune response that could be elicited by invertase-generated free monosaccharides in the apoplast, is likely to be circumvented [93]. The up-regulation of *srt1* already on the hydrophobic surface suggests that uptake of sucrose may already be important during the early stages of colonization.

Remarkably, two P-type ATPases (*acu1* and *acu2*) were also highly induced during filament and appressorium formation (Table S6) and also in tumor tissue [21]. Acu1 and Acu2 of *U. maydis* are high affinity potassium and sodium transporters [94]. We hypothesized that Acu1 and Acu2 might play a role in potassium and/or sodium supply during pathogenic development and deleted the genes in the solopathogenic AN1 strain. The Δ*acu1* single mutant and the Δ*acu1*Δ*acu2* double mutant were unable to grow on sodium/potassium starvation media while Δ*acu2* single mutants showed normal growth under those conditions (Figure S8). The growth defect of the Δ*acu1* and Δ*acu1*Δ*acu2* strains was fully suppressed by adding potassium to the medium but not by adding sodium (Figure S8). This demonstrates that *acu1* is essential for potassium supply under starvation conditions. However, neither the *acu1* and *acu2* single deletion mutants nor the double mutant were affected in their ability to form filaments and appressoria, and their virulence was comparable to the AN1 strain (Figure S3). This indicates that at least under our laboratory conditions, high affinity potassium uptake of *U. maydis* is dispensable for the biotrophic interaction.

The genes for putative mechano-sensitive ion channel transporters (*um10559*, *um04550* and *um02237*) were also up-regulated during filament formation. Since mechano-sensitive ion channels are thought to function in surface perception in fungi [3], [95], it will be interesting to investigate the role of these genes for pathogenic development by deleting them simultaneously.

### Transcription factors act as key regulators of differentiation

The switch from saprophytic growth to pathogenic development requires a precise regulation of cellular and developmental processes. The key elements in such processes are transcription factors (TFs). Of a total of 55 differentially regulated TFs, 28 were induced during filament and appressorium formation and 9 of them showed a particularly high expression in appressoria compared to filaments (HS+FA vs GC; Table S7). These included 4 uncharacterized Zn_2_Cys_6_ TFs (*um03682*, *um04999*, *um12189* and *um04242*), designated *aiz1*–*aiz4* (appressorium induced Zn_2_Cys_6_). Zn_2_Cys_6_ TFs are unique to fungi, and in *Fusarium spp.* TFs of this type have important functions for penetration and colonization of plants [96], [97]. However, *U. maydis* deletion mutants for *aiz1*, *aiz2* and *aiz3* exhibited no significant differences to the AN1 progenitor strain with respect to filamentation, appressorium formation and virulence (Figure S3). *aiz4* (*um04242*) most likely encodes an essential function, as it was impossible to obtain deletion mutants in the AN1 background. To prove that the generated deletion construct is functional we performed the gene replacement in the diploid strain FBD11 [98]. Here we obtained viable strains with one copy of *aiz4* deleted (not shown), providing strong indication that *aiz4* is indeed essential.

**Figure 4:**
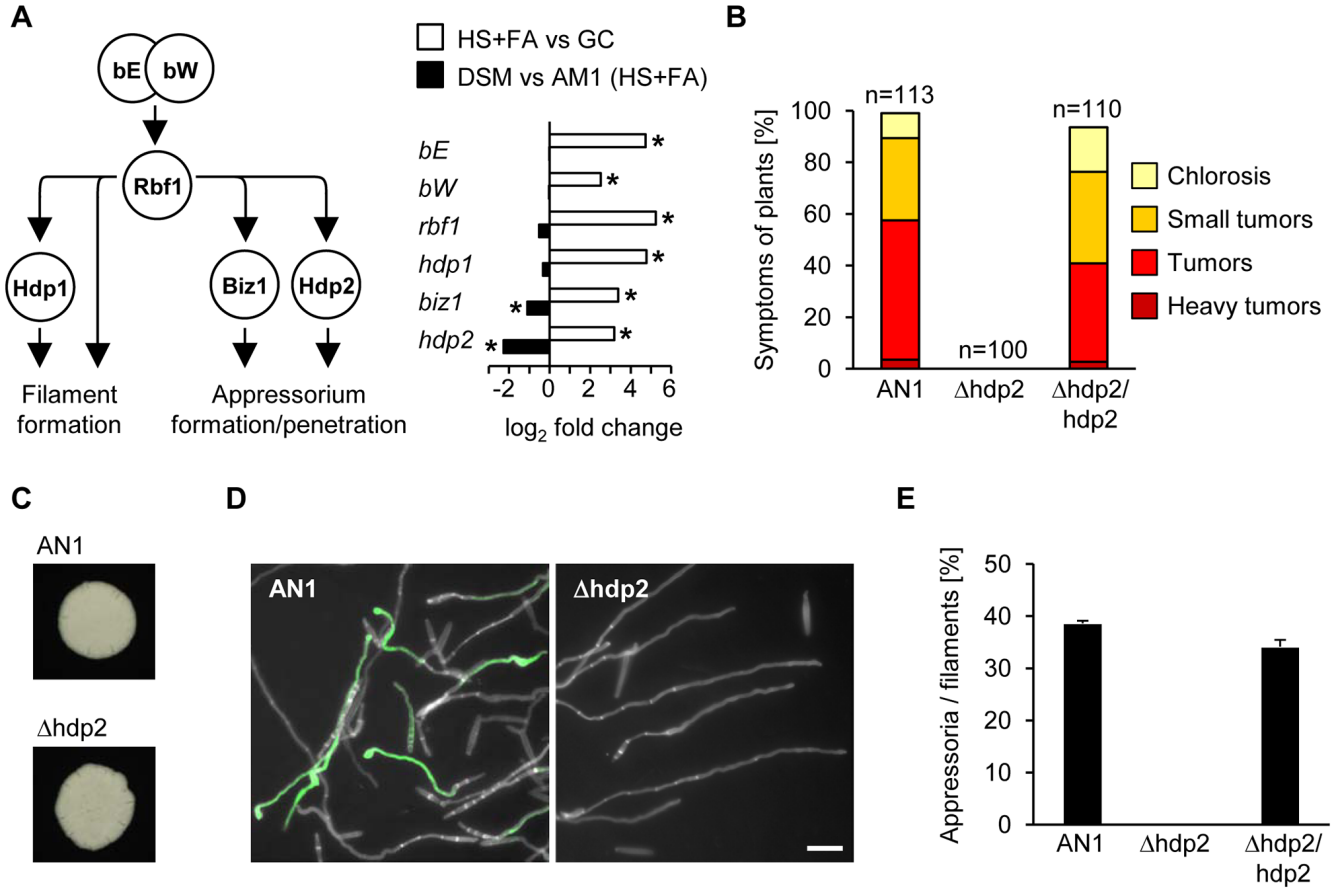
Sho1 and Msb2 regulate the expression of the appressorium-specific transcription factors *biz1* and *hdp2*. **A**. Schematic overview of the transcriptional *b*-network (left) and its regulation during appressorium formation (right). All components of the *b*-cascade are significantly up-regulated in response to plant surface cues (HS+FA vs GC). Only appressorium-specific *biz1* and *hdp2* genes are down-regulated in AM1Δsho1Δmsb2 mutants compared to the AM1 control strain (DSM vs AM1). *Significant difference (see Materials and Methods for details) HS: Hydrophobic surface, FA: Fatty acid, GC: Glass control **B**. Symptoms caused by AN1, AN1Δhdp2 and the complemented strain AN1Δhdp2/hdp2. Strains were injected into maize seedlings and symptoms were scored 12 days after infection according to severity; the color code for each category is given below. Three independent experiments were carried out and the average values are expressed as a percentage of the total number of infected plants (n), which is given above each column. **C**. Filament formation. The indicated strains were spotted on PD charcoal plates and incubated for 24 h at 28°C. The white fuzzy colonies reflect the formation of *b*-dependent filaments. **D**. Appressorium formation. AN1 and the indicated derivatives were sprayed on ParafilmM with 100 µM HDA and incubated for 18 h at 28°C. Hyphae were stained with calcofluor and GFP fluorescence was monitored. Scale bar represents 10 µm. **E**. Quantification of appressoria from the indicated strains using the same conditions as in D. Average percentage of cells that expressed the appressoria-specific GFP-reporter was determined relative to the cells that had formed filaments. In three independent experiments more than 400 cells were analyzed and error bars indicate standard error.

In *U. maydis*, the heterodimeric transcriptional activator bE/bW, is induced in response to pheromone perception [99], [100]. The bE/bW complex serves as molecular switch for pathogenic development and induces a hierarchical network of downstream TFs (Figure 4A). This was uncovered by studying in a time-resolved manner the consequences of induced over-expression of a bE/bW heterodimer [31]. From the 206 *b*-dependently induced genes identified in this study [31], 145 genes were found induced by the surface cues, and from the 139 *b*-dependently repressed genes, 78 genes were repressed by the plant-derived surface signals (Table S1). Thus, the majority of the *b*-induced genes were also induced during appressorium formation, including all TFs of the *b*-cascade (*bE*, *bW*, *rbf1*, *hdp1*, *hdp2* and *biz1*; Figure 4A). Interestingly, two of the downstream TFs, *biz1* and *hdp2*, were *sho1* and *msb2*-dependently expressed while the upstream TFs *rbf1*, *bE* and *bW* as well as the downstream TF *hdp1* did not require *sho1* and *msb2* for their induction (Figure 4A). Preliminary analysis showed that deletion mutants for *hdp2* were nonpathogenic [31] and we now demonstrated that such mutants are unable to form appressoria, while filamentous growth was not affected (Figure 4B–E). Both defects could be fully complemented by introducing a single copy of *hdp2* (Figure 4B and E). *hdp2* mutants thus resemble *sho1 msb2* double mutants with respect to appressorium formation and virulence. For *biz1* previous studies also demonstrated a specific role during appressorial penetration [32]. To determine whether *hdp2* and *biz1* are downstream targets of Sho1 and Msb2, we performed qPCR to measure the expression of several *sho1*/*msb2*-regulated genes in *hdp2* and *biz1* deletion mutants after spraying on HS with FA. All tested genes, i.e. the effector genes *pit2* and *cmu1*, the appressoria marker gene *am1*, and the CWDE genes *afg1* and *egl2* showed reduced expression in the *hdp2* mutant that was comparable to the expression level in *sho1 msb2* mutants (Figure S9). Conversely, most of the genes were normally expressed in *biz1* mutants, except for *egl2* which was *biz1*-dependently induced (Figure S9). These data indicate that the *b*-cascade components *hdp2* and *biz1* function downstream of *sho1* and *msb2* and that *hdp2* may be their primary target.

Another known transcriptional target of the *b*-cascade is the MAP kinase *kpp6* [31], [35]. This MAP kinase regulates the ability of appressoria to successfully penetrate. Our data showed that also *kpp6* is *sho1* and *msb2*-dependently expressed. This induction is likely to constitute a positive feedback loop to enhance MAP kinase activity and finally force plant penetration.

Interestingly, the induction of *hdp2*, *biz1* and *kpp6* was not completely abolished in the Δ*sho1*Δ*msb2* mutant. We consider it likely that this remaining induction is caused by *b*-cascade activity stimulated by pheromone signaling. This is supported by the up-regulation of the pheromone response factor *prf1* during filament formation. Pheromone signaling has been previously shown to be part of the response to the hydrophobic surface [34]. Thus, the role of Sho1 and Msb2 is to feed into the *b*-cascade downstream of bE/bW to specifically enhance the expression of those genes that are responsible for the formation and function of appressoria. One possibility is that the *b*-cascade is redirected at the level of the Rbf1 regulator. However, this is unlikely because mutation of all six putative MAP kinase phosphorylation sites in Rbf1 does not influence virulence (D. Lanver and R. Kahmann, unpublished). Therefore, we speculate that the link between Sho1 and Msb2 and induction of the TFs is established by a yet unidentified TF that works in parallel with Rbf1 to induce expression of *hdp2*, *biz1*, and *kpp6*.

### Summary and Conclusions

Our study has highlighted that almost 20% of all *U. maydis* genes are differentially regulated under the *in vitro* conditions that induce appressoria. Among the surface cue-induced genes we detected a strong over-representation of genes encoding secreted proteins, e.g. CWDEs that facilitate plant penetration. Furthermore, secreted effectors that are specifically needed for the establishment of biotrophy after entering the plant were transcriptionally induced. This expression pattern demonstrates that *U. maydis* becomes prepared to suppress plant defense responses already before penetration. Given the narrow host range of *U. maydis* establishing a compatible interaction only with maize and its ancestor teosinte, it is intriguing that compatibility factors such as effector genes are induced by the unspecific plant surface cues hydrophobicity and hydroxy fatty acid. This suggests that under natural conditions *U. maydis* and probably other smut fungi use trial and error to find a compatible host plant. This idea is supported by a recent study that demonstrates efficient appressorium formation of *U. maydis* on the non-host plant barley [101].

Compared to the extensive transcriptional response induced by the plant surface cues hydrophobicity and hydroxy fatty acids, two putative sensors of these cues, Sho1 and Msb2, are involved in the induction of only a subset of those genes. While it is not exactly clear what Sho1 and Msb2 sense, our transcriptomic data now provides evidence that Sho1 and Msb2 in *U. maydis* are required specifically for the expression of essential virulence factors. It is conceivable that during growth on a hydrophobic leaf surface the function of Sho1 and Msb2 is to determine the site for penetration and to trigger at this stage the local expression of plant cell wall degrading enzymes and effectors. Sho1 and Msb2 are likely to exert their effects on these genes via the *b*-cascade. They contribute specifically to full induction of the transcriptional *b*-network by regulating only those TFs that have their function during appressorium development. Thus, Sho1 and Msb2 direct the central transcriptional network towards penetration.

## Materials and Methods

### *U. maydis* strains and growth conditions

The solopathogenic SG200 strain, its derivative AM1, which carries the appressorium-specific GFP-reporter construct in the *ip* locus, and the AM1Δsho1Δmsb2 strain have been described previously [21], [34], [36].

*U. maydis* strains were grown in liquid YEPSL (0.4% yeast extract, 0.4% peptone, 2% sucrose) or on solid potato dextrose (PD) plates at 28°C. For filament induction, PD plates containing 1% activated charcoal were used [102]. For growth assays, strains were grown to an OD_600_ of 0.8 in YEPSL, the OD was adjusted to 1.0 in water and serial 1∶10 dilutions were spotted on CM glucose medium [102]. This CM medium was supplemented with 1 mM H_2_O_2_ to generate oxidative stress or 70 µg/ml congo red (Sigma, Germany) to generate cell wall stress. For potassium/sodium starvation conditions, strains were spotted on arginine phosphate (AP) medium [103].

### Construction of *U. maydis* strains

To construct AN1, a strain identical to AM1 except for the resistance marker and the insertion site of the appressorial marker construct, the 1.8 kb carboxin resistance cassette of pAM1 [34] was replaced with the 1.4 kb nourseothricin resistance cassette from pMF1-n [104]. The resulting plasmid pAN1 was linearized with SacI and transformed in the SG200 strain to allow ectopic integration of the appressorial marker construct. The selected AN1 transformant was as virulent as the AM1 strain and GFP fluorescence in appressoria was comparable to AM1 (not shown).

For gene disruptions, a PCR-based strategy described in [105] and the SfiI insertion cassette system [104], [106] were used. For each deletion construct 1 kb of the left border (lb) and the right border (rb) were PCR-amplified and ligated either with hygromycin, geneticin, or nourseothricin resistance cassette via SfiI restriction sites. The resulting fragments were either PCR-amplified and transformed into *U. maydis* protoplasts or cloned into pCRII-TOPO (Invitrogen) and transformed as linearized plasmids. All gene replacements were verified by Southern blot analysis.

For complementation analysis the respective genes were cloned into p123 [107]. This plasmid integrates into the *U. maydis ip* locus and mediates carboxin resistance. The *acu1*, *acu2*, *afg1*, *afg2* and *afg3* genes were amplified including 2 kb of their upstream sequence and integrated into p123 via NdeI/NotI sites. The respective plasmids are termed pP_acu1_:acu1, pP_acu2_:acu2, pP_afg1_:afg1, pP_afg2_:afg2 and pP_afg3_:afg3. The *ukc2* gene was also amplified with 2 kb upstream sequence and cloned into p123 with Acc65I/NotI to yield plasmid pP_ukc2_:ukc2 Expression of these genes is controlled by their native promoters and the *nos*-terminator. The *hdp2* complementation construct pP_hdp2_:hdp2:T_hdp2_ contained 2.5 kb of the upstream sequence and 0.4 kb of the downstream terminator sequence integrated with NdeI/SbfI sites into NdeI/NsiI-cleaved p123. In this context *hdp2* is under the control of its own promoter and terminator. All strains and primers used in this study are listed in Table S8 and S9, respectively.

### Virulence assay and quantification of appressoria and penetration events

For virulence assays, solopathogenic *U. maydis* strains were grown in YEPSL medium to an OD_600_ of 0.8 and concentrated in H_2_O to a final OD_600_ of 1.0. This suspension was syringe-inoculated into seven-day-old maize seedlings of the variety Early Golden Bantam (Olds Seeds, Madison). Disease symptoms were evaluated after 12 days according to the disease rating criteria reported in [21]. An illustration of typical disease symptoms of the infected plants is given in Figure S2. Quantification of *in vitro* appressorium formation and quantification of penetration events on maize leaves was done as described previously [36], [108].

### RNA isolation from filaments and appressoria

AM1 and AM1Δsho1Δmsb2 strains were grown to an OD_600_ of 0.8 in YEPSL, concentrated to an OD_600_ of 1.0 in low nutrient medium (2% YEPSL) and supplemented with or without 100 µM (f.c.) 16-hydroxy hexadecanoic acid (HDA, Sigma, Germany). 2 ml cell suspensions were sprayed (EcoSpray, Roth, Germany) on 100 cm^2^ ParafilmM and incubated at 100% humidity at 28°C for 12 h. As control 2 ml cell suspensions were sprayed on glass plates and incubated for 2 h at 28°C. Cells that were not attached to the hydrophobic surface were washed away with water. Attached filaments and appressoria were harvested using a cell scraper (Greiner, Germany) and 1 ml of a 1∶1 mixture of aqua-phenol/chloroform and AE-buffer (50 mM NaAc, 10 mM EDTA, pH 5.3). From glass plates, where cells do not attach, cells were directly transferred to the aqua-phenol/chloroform/AE-buffer mixture. Samples were vortexed for 15 min with glass beads and incubated for 10 min at 60°C. Samples were centrifuged (15 min, 16,000 g) and the supernatant was washed with aqua-phenol. After additional centrifugation, RNA was precipitated with 300 mM (f.c.) sodium acetate and 2.5 volumes of ethanol. RNA was pelleted (10 min, 16,000 g), washed in ethanol, air-dried and dissolved in water. Finally, RNA was purified by using Ambion Turbo DNA free Kit (Life Technologies, Carlsbad/USA) and RNeasy Mini Kit (Qiagen, Hilden/Germany). The RNA quality was controlled using an Agilent 2100 Bioanalyzer.

### Microarray analysis

Transcriptional profiling was performed with Affymetrix *Ustilago maydis* Custom GeneChips (MPIUstilagoA) [109]. Probe sets for the individual genes are visualized at http://mips.helmholtz-muenchen.de/genre/proj/ustilago/. All experiments were done in three biological replicates.

The GeneChip 3′ IVT Express Kit (Affymetrix, High Wycombe/UK) was used for one-step amplification of 100 ng purified total RNA and each GeneChip was hybridized with 15 µg of the fragmented aRNA using standard Affymetrix protocols (Euk2V3 protocol on GeneChip Fluidics Station 450). The arrays were scanned (Affymetrix GSC3000) and the resulting image data analyzed with Affymetrix GeneChip Operating Software (GCOS; Affymetrix Microarray Suite 5.9) as described previously [109]. The values of individual probes belonging to one probe set were averaged and normalized using Partek Genomics Suite 6.5 (Partek Inc., St. Louis, MO, USA). The average fluorescence intensity of all annotated genes was calculated using the Robust Multiarray Analysis (RMA) algorithm [110]. To identify differentially expressed genes between the different experimental groups, a one-way analysis of variance (ANOVA) [111] was performed. The resulting p-values were corrected for multiple-testing with a false discovery rate procedure (FDR) [112]. Criteria for significance were a p-value (per sample) of 0.05 with a FDR of 0.01 and a fold-change of ≥2. Expression data were submitted to GeneExpressionOmnibus (http://www.ncbi.nlm.nih.gov/geo/) under the accession number GSE53947.

### Quantitative real-time PCR

Expression of individual genes was analyzed by qPCR. 10 ng of isolated total RNA was reverse-transcribed using First-Strand cDNA Synthesis Kit (Fermentas, St. Leon-Roth/Germany). qPCR was performed on a Bio-Rad iCycler using undiluted cDNA and SYBR Green qPCR SuperMix-UDG (Invitrogen, Karlsruhe/Germany). Cycling conditions were 2 min 95°C, followed by 45 cycles of 30 s 95°C/30 s 62°C/30 s 72°C. The peptidylprolyl isomerase (*ppi*) *um03726.2* served as reference gene. Relative expression was determined using the ΔΔCt method [113]. All qPCR primers are listed in Table S9.

### Accession numbers

*sho1* (*um03156*) XM_754210, *msb2* (*um00480*) XM_751534, *afg1* (*um01829*) XM_752883, *afg2* (*um00837*) XM_751891, *afg3* (*um04309*) XM_755363, *egl1* (*um06332*) XM_757386, *egl2* (*um02523*) XM_753577, *egl3* (*um04816*) XM_755870, *ukc2* (*um02741*) XM_753795, *gad1* (*um06063*) XM_757117, *gatA* (*um01080*) XM_752134, *acu1* (*um06433*) XM_757487, *acu2* (*um05038*) XM_756092, *aiz1* (*um03682.2*) XM_754736, *aiz2* (*um04999*) XM_756053, *aiz3* (*um12189*) XM_753443, *aiz4* (*um04242*) XM_755296, *hdp2* (*um04928*) XM_755982, *biz1* (*um02549*) XM_753603. Additional accession numbers are listed in Table S1 and S2.

## Supporting Information

Figure S1 **Validation of microarray data with quantitative real-time PCR.** Verification of gene induction by qPCR of selected genes shown to be induced by HS vs GC and HS+FA vs GC in the microarray analysis. The AM1 strain was sprayed on ParfilmM and incubated for 12 h. Relative expression after growth on the glass control surface (GC) was set to 1 for each gene. The fold change expression on hydrophobic surface (HS) alone and with addition of hydroxy fatty acid (HS+FA) is shown. In the microarray analysis *um12189*, *um06332*, *um06433* and *um05038* were significantly induced by HS alone and by the combination of HS and FA, while *um02104.2* and *um01829* were significantly induced only by the combination of both stimuli. Expression pattern in qPCR data correlates with microarray data. Error bars denote standard error of three replicates. *Significant difference to the respective glass control (p<0.05, student's t).
(TIF)

Figure S2 **Qualitative disease rating criteria for** ***U. maydis*** **infections.** The scheme shows representative *U. maydis*-infected maize plants for the different disease ratings. These categories representing chlorosis (usually accompanied by anthocyanin induction; yellow), small tumors (dark yellow), tumors (red) and heavy tumors (dark red) are used throughout this study to quantify virulence of respective *U. maydis* strains.
(TIF)

Figure S3 **Virulence of selected** ***U. maydis*** **deletion mutants.** **A**. AN1, SG200 and the indicated derived strains were spotted on PD charcoal plates and incubated for 24 h at 28°C. The white fuzzy colonies reflect the formation of *b*-dependent filaments. **B**. The indicated strains were sprayed on ParafilmM with 100 µM HDA and incubated for 18 h at 28°C. The average percentage of filaments that formed appressoria was determined. More than 400 filaments per strain were analyzed in three independent experiments. Error bars indicate standard error. **C**. The indicated strains were injected into maize seedlings and symptoms were scored 12 days after infection according to severity; the color code for each category is given on the right. Three independent experiments were carried out and the average values are expressed as a percentage of the total number of infected plants (n), which is given to the right of each bar. According to student's t test differences in virulence to the respective progenitor strain were not significant (p>0.05).
(TIF)

Figure S4 **Expression pattern of arabinofuranosidase genes during development on hydrophobic surface plus/minus hydroxy fatty acid.** The AM1 strain was sprayed on ParafilmM and incubated for 12 h. Relative expression was determined by qPCR for *afg1*, *afg2* and *afg3*. Expression after growth on the glass control surface (GC) was set to 1 for each gene. The fold change expression on hydrophobic surface alone (HS) and with addition of hydroxy fatty acid (HS+FA) is shown. The experiment was conducted in three biological replicates and error bars denote standard error. *Significant difference (p<0.05, student's t).
(TIF)

Figure S5 **The NDR kinase Ukc2 is required for pathogenic development of** ***U. maydis*** **.** **A**. Virulence of AN1, AN1Δukc2 and the complemented strain AN1Δukc2/ukc2. Strains were injected into maize seedlings and symptoms were scored 12 days after infection, the color code for each disease category is given below. Three independent experiments were carried out and the average values are expressed as a percentage of the total number of infected plants (n), which is given above each column. *Significant difference for each category and pair given above (p<0.05, student's t) ns: not significant **B**. Filament formation. The indicated strains were spotted on PD charcoal plates and incubated for 24 h at 28°C. The white fuzzy colonies reflect the formation of *b*-dependent filaments. **C**. Appressorium formation. AN1 and the indicated derivatives were sprayed on ParafilmM with 100 µM HDA and incubated for 18 h at 28°C. Hyphae were stained with calcofluor and the average percentage of cells that expressed the appressorial GFP-marker was determined relative to the cells that had formed filaments. In three independent experiments more than 400 filaments were analyzed and error bars indicate standard error. *Significant difference (p<0.05, student's t). **D**. The strains were grown to mid log phase and serial dilutions were spotted on CM and CM supplemented with 70 µg/ml congo red. Plates were incubated for 2 days (CM) and 3 days (congo red) at 28°C.
(TIF)

Figure S6 **Functional categories of differentially regulated genes during development on hydrophobic surface plus/minus hydroxy fatty acid.** Differentially regulated genes during filament formation (HS vs GC), appressorium formation (HS+FA vs GC) and the transition from filaments to appressoria (HS+FA vs HS) were grouped into functional categories using FunCatDB. Functional groups containing more than 30 genes that were significantly enriched in at least one of the respective gene sets are depicted. Black bars represent the number of genes detected in the gene set and white bars represent the number of genes expected by chance (calculated using the functional distribution of all predicted *U. maydis* genes) *, ** and *** denote p-values (hypergeometric distribution) of p<0.01, p<0.001 and p<0.0001, respectively. HS: Hydrophobic surface, FA: Fatty acid, GC: Glass control.
(TIF)

Figure S7 ***gatA*** **and** ***gad1*** **of** ***U. maydis*** **are not required for oxidative stress response.** The indicated strains were grown to mid log phase and serial dilutions were spotted on CM and CM supplemented with 1 mM H_2_O_2_. Plates were incubated for 2 days at 28°C.
(TIF)

Figure S8 ***acu1*** **is required for potassium homeostasis under starvation conditions.** AN1 and the indicated derived strains were grown to mid log phase and serial dilutions were spotted on sodium/potassium-starved AP (arginine phosphate) medium, and AP medium supplemented with either 5 mM KCl or NaCl. Plates were incubated for 3 days at 28°C. The reduced growth of the *acu1* complemented strain most likely results from over-expression of *acu1* as this strain carries multiple *acu1* copies.
(TIF)

Figure S9 **Expression of genes encoding secreted proteins in** ***hdp2*** **and** ***biz1*** **mutants.** The indicated strains were sprayed on ParafilmM with 100 µM HDA and incubated for 12 h. Relative expression was determined by qPCR for the appressoria marker gene *am1*, the effector genes *pit2* and *cmu1*, and the CWDE genes *afg1* and *egl2*. For each gene the expression in the AM1 strain was set to 1 and the ratio to the expression in Δ*hdp2*, Δ*biz1* and Δ*sho1*Δ*msb2* strains calculated. The experiment was conducted in three replicates and the same scale is used for all genes. Error bars denote standard error. *Significant difference (p<0.05, student's t).
(TIF)

Table S1 **Differentially regulated genes in** ***U. maydis*** **AM1 strain during development on hydrophobic surface plus/minus hydroxy fatty acid.**
(XLSX)

Table S2 **Differentially regulated genes in AM1Δsho1Δmsb2 mutant compared to AM1 during development on hydrophobic surface plus/minus hydroxy fatty acid.**
(XLSX)

Table S3 **Genes encoding secreted proteins differentially regulated during development on hydrophobic surface plus/minus hydroxy fatty acid.**
(XLSX)

Table S4 **Expression profile of all genes encoding secreted carbohydrate active enzymes (CAZy) under** ***in vitro*** **conditions inducing filaments and appressoria.**
(XLSX)

Table S5 **Expression profile of genes encoding enzymes of primary metabolism under** ***in vitro*** **conditions inducing filaments and appressoria.**
(XLSX)

Table S6 **Genes encoding plasma membrane transporters differentially regulated during development on hydrophobic surface plus/minus hydroxy fatty acid.**
(XLSX)

Table S7 **Genes encoding transcription factors differentially regulated during development on hydrophobic surface plus/minus hydroxy fatty acid.**
(XLSX)

Table S8 ***U. maydis*** **strains used in this study.**
(DOCX)

Table S9 **Primer used in this study.**
(XLSX)
