# Supplementary figures and images for: Plant Surface Cues Prime Ustilago maydis for Biotrophic Development
Source: PLoS Pathog. 2014 Jul 17;10(7):e1004272. doi: 10.1371/journal.ppat.1004272 (PMC4102580; doi:10.1371/journal.ppat.1004272)

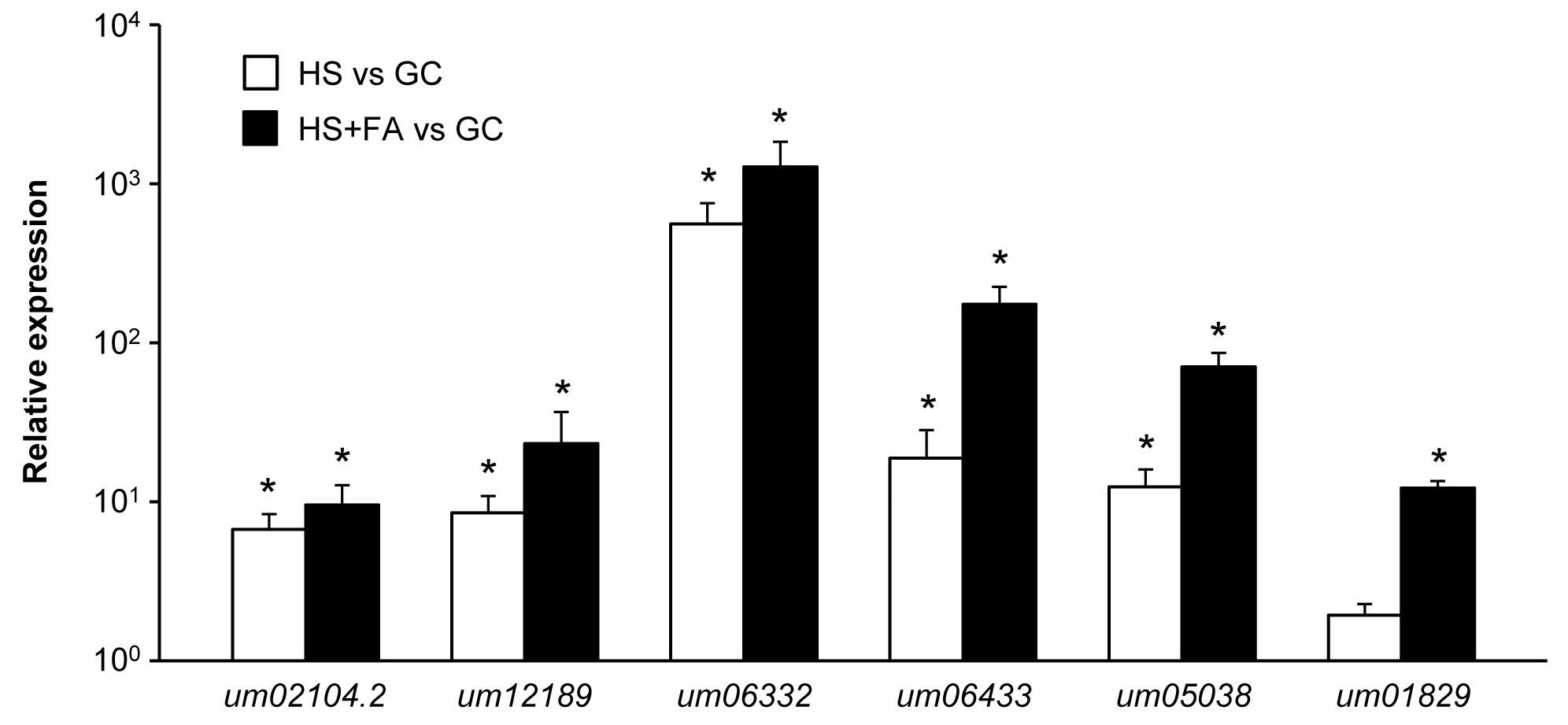

Supplement: Figure S1 — Validation of microarray data with quantitative real-time PCR. Verification of gene induction by qPCR of selected genes shown to be induced by HS vs GC and HS+FA vs GC in the microarray analysis. The AM1 strain was sprayed on ParfilmM and incubated for 12 h. Relative expression after growth on the glass control surface (GC) was set to 1 for each gene. The fold change expression on hydrophobic surface (HS) alone and with addition of hydroxy fatty acid (HS+FA) is shown. In the microarray analysis um12189, um06332, um06433 and um05038 were significantly induced by HS alone and by the combination of HS and FA, while um02104.2 and um01829 were significantly induced only by the combination of both stimuli. Expression pattern in qPCR data correlates with microarray data. Error bars denote standard error of three replicates. *Significant difference to the respective glass control (p<0.05, student's t). (TIF) [file ppat.1004272.s001.tif]

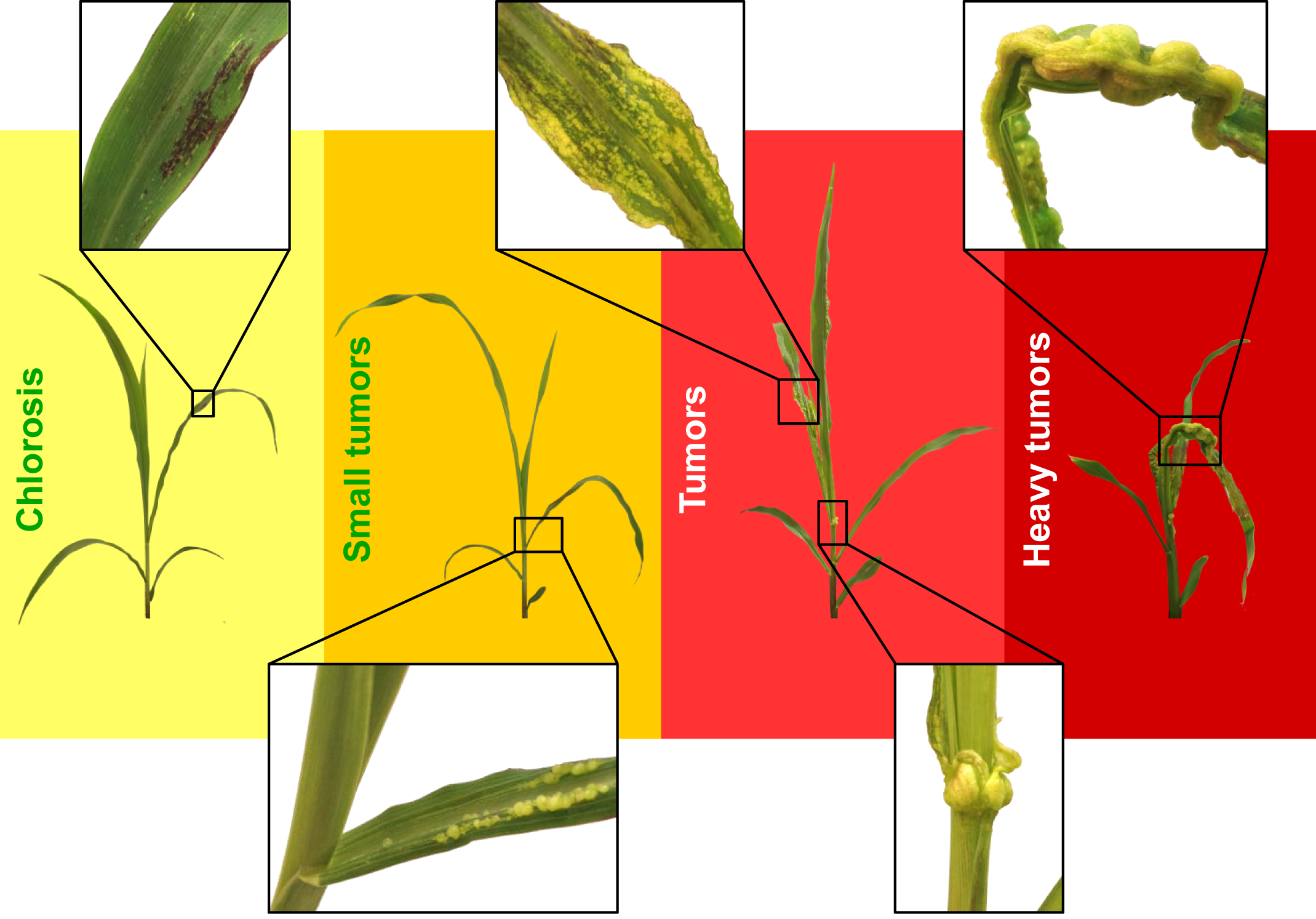

Supplement: Figure S2 — Qualitative disease rating criteria for U. maydis infections. The scheme shows representative U. maydis-infected maize plants for the different disease ratings. These categories representing chlorosis (usually accompanied by anthocyanin induction; yellow), small tumors (dark yellow), tumors (red) and heavy tumors (dark red) are used throughout this study to quantify virulence of respective U. maydis strains. (TIF) [file ppat.1004272.s002.tif]

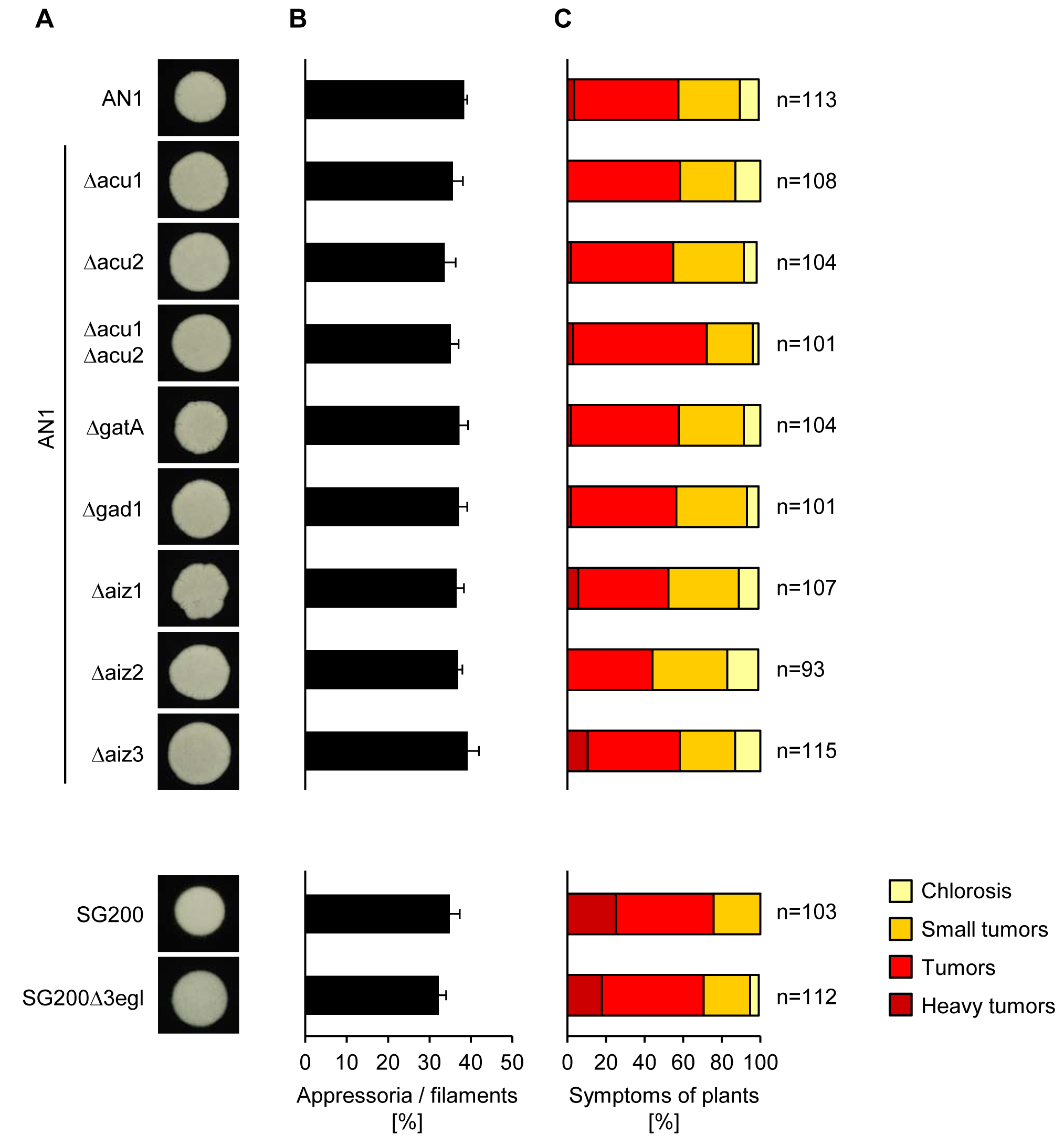

Supplement: Figure S3 — Virulence of selected U. maydis deletion mutants. A. AN1, SG200 and the indicated derived strains were spotted on PD charcoal plates and incubated for 24 h at 28°C. The white fuzzy colonies reflect the formation of b-dependent filaments. B. The indicated strains were sprayed on ParafilmM with 100 µM HDA and incubated for 18 h at 28°C. The average percentage of filaments that formed appressoria was determined. More than 400 filaments per strain were analyzed in three independent experiments. Error bars indicate standard error. C. The indicated strains were injected into maize seedlings and symptoms were scored 12 days after infection according to severity; the color code for each category is given on the right. Three independent experiments were carried out and the average values are expressed as a percentage of the total number of infected plants (n), which is given to the right of each bar. According to student's t test differences in virulence to the respective progenitor strain were not significant (p>0.05). (TIF) [file ppat.1004272.s003.tif]

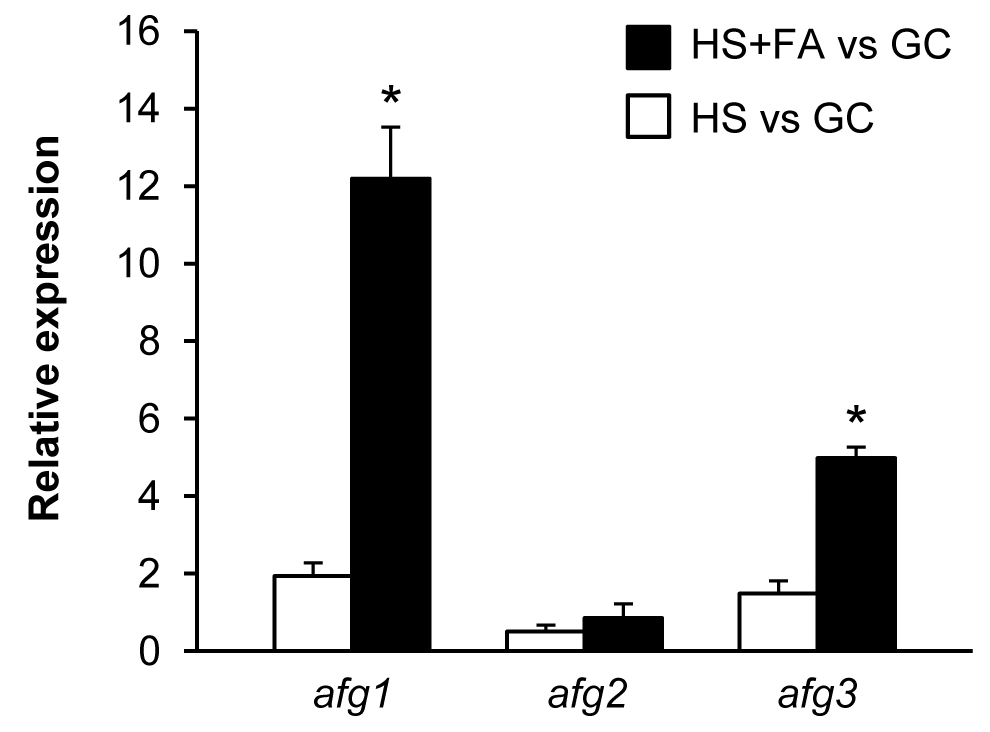

Supplement: Figure S4 — Expression pattern of arabinofuranosidase genes during development on hydrophobic surface plus/minus hydroxy fatty acid. The AM1 strain was sprayed on ParafilmM and incubated for 12 h. Relative expression was determined by qPCR for afg1, afg2 and afg3. Expression after growth on the glass control surface (GC) was set to 1 for each gene. The fold change expression on hydrophobic surface alone (HS) and with addition of hydroxy fatty acid (HS+FA) is shown. The experiment was conducted in three biological replicates and error bars denote standard error. *Significant difference (p<0.05, student's t). (TIF) [file ppat.1004272.s004.tif]

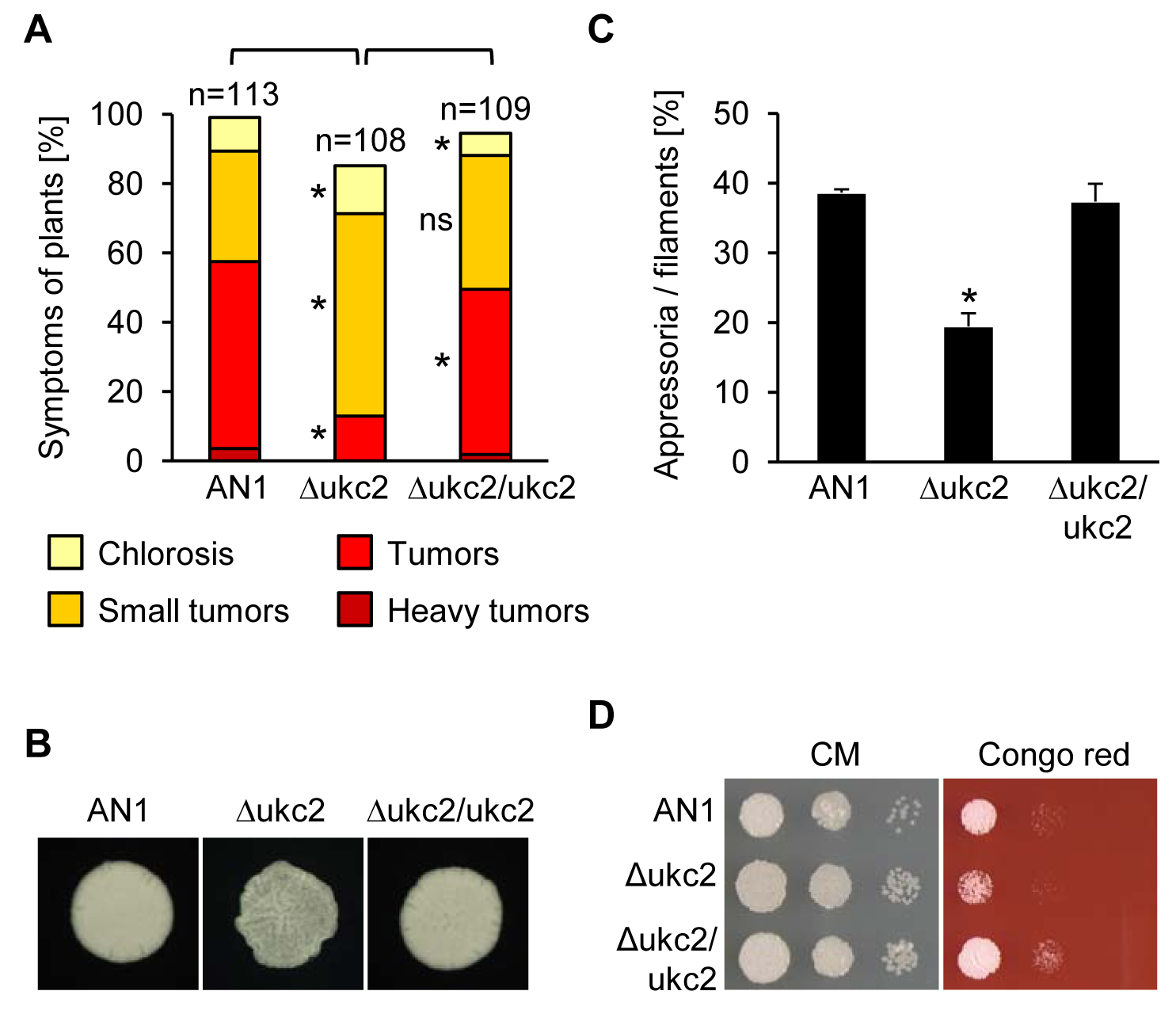

Supplement: Figure S5 — The NDR kinase Ukc2 is required for pathogenic development of U. maydis . A. Virulence of AN1, AN1Δukc2 and the complemented strain AN1Δukc2/ukc2. Strains were injected into maize seedlings and symptoms were scored 12 days after infection, the color code for each disease category is given below. Three independent experiments were carried out and the average values are expressed as a percentage of the total number of infected plants (n), which is given above each column. *Significant difference for each category and pair given above (p<0.05, student's t) ns: not significant B. Filament formation. The indicated strains were spotted on PD charcoal plates and incubated for 24 h at 28°C. The white fuzzy colonies reflect the formation of b-dependent filaments. C. Appressorium formation. AN1 and the indicated derivatives were sprayed on ParafilmM with 100 µM HDA and incubated for 18 h at 28°C. Hyphae were stained with calcofluor and the average percentage of cells that expressed the appressorial GFP-marker was determined relative to the cells that had formed filaments. In three independent experiments more than 400 filaments were analyzed and error bars indicate standard error. *Significant difference (p<0.05, student's t). D. The strains were grown to mid log phase and serial dilutions were spotted on CM and CM supplemented with 70 µg/ml congo red. Plates were incubated for 2 days (CM) and 3 days (congo red) at 28°C. (TIF) [file ppat.1004272.s005.tif]

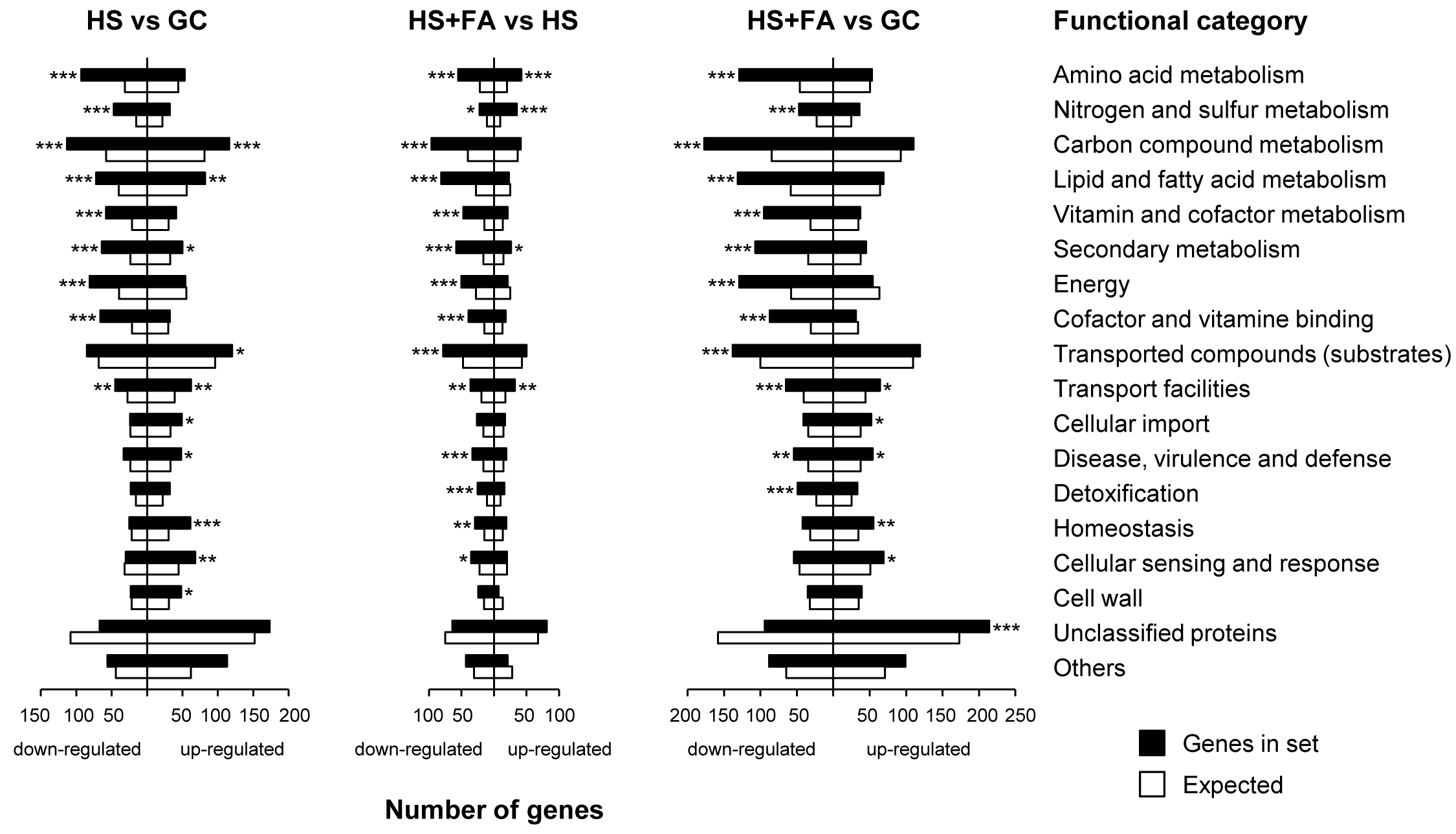

Supplement: Figure S6 — Functional categories of differentially regulated genes during development on hydrophobic surface plus/minus hydroxy fatty acid. Differentially regulated genes during filament formation (HS vs GC), appressorium formation (HS+FA vs GC) and the transition from filaments to appressoria (HS+FA vs HS) were grouped into functional categories using FunCatDB. Functional groups containing more than 30 genes that were significantly enriched in at least one of the respective gene sets are depicted. Black bars represent the number of genes detected in the gene set and white bars represent the number of genes expected by chance (calculated using the functional distribution of all predicted U. maydis genes) *, ** and *** denote p-values (hypergeometric distribution) of p<0.01, p<0.001 and p<0.0001, respectively. HS: Hydrophobic surface, FA: Fatty acid, GC: Glass control. (TIF) [file ppat.1004272.s006.tif]

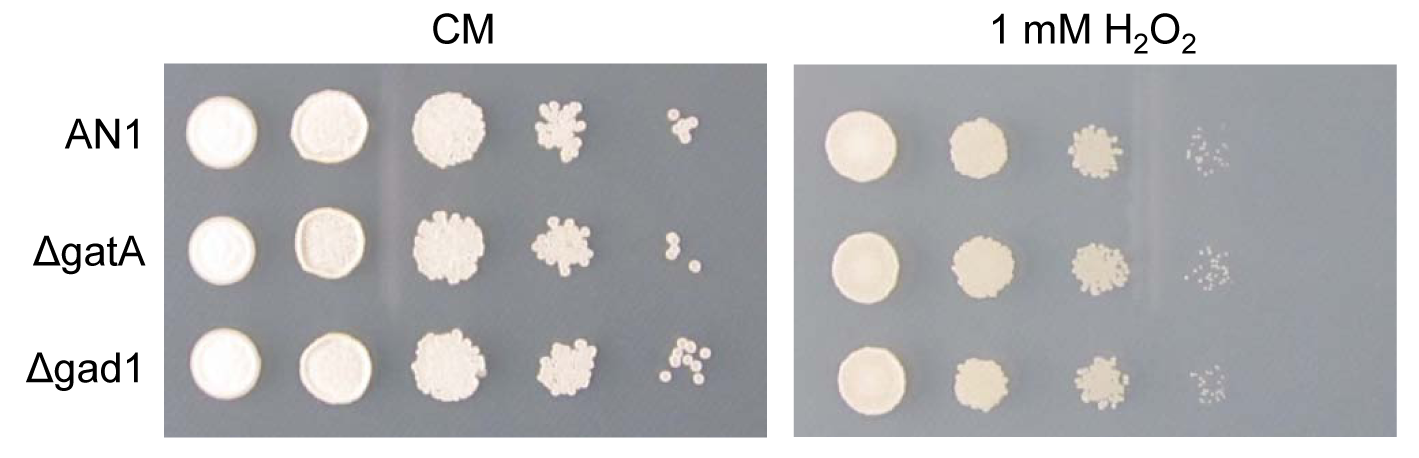

Supplement: Figure S7 — gatA and gad1 of U. maydis are not required for oxidative stress response. The indicated strains were grown to mid log phase and serial dilutions were spotted on CM and CM supplemented with 1 mM H2O2. Plates were incubated for 2 days at 28°C. (TIF) [file ppat.1004272.s007.tif]

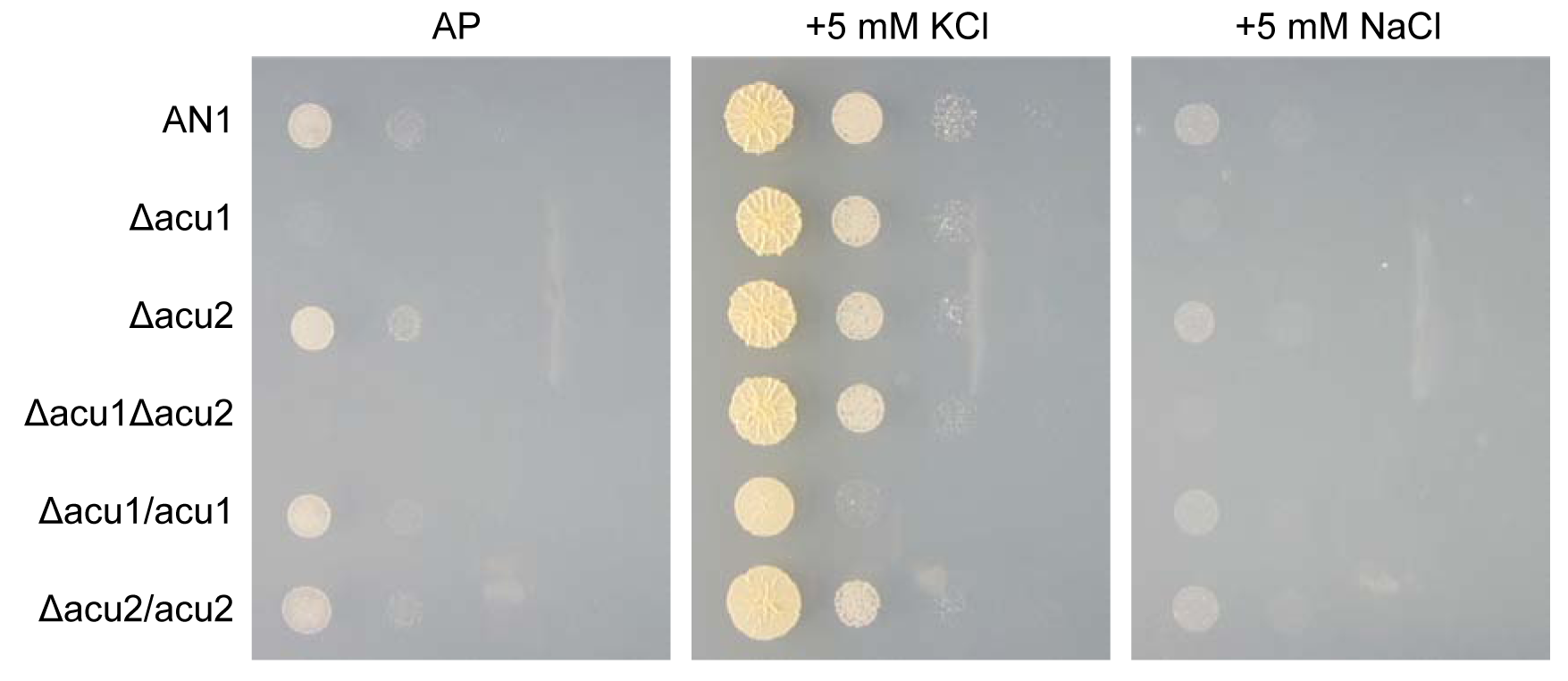

Supplement: Figure S8 — acu1 is required for potassium homeostasis under starvation conditions. AN1 and the indicated derived strains were grown to mid log phase and serial dilutions were spotted on sodium/potassium-starved AP (arginine phosphate) medium, and AP medium supplemented with either 5 mM KCl or NaCl. Plates were incubated for 3 days at 28°C. The reduced growth of the acu1 complemented strain most likely results from over-expression of acu1 as this strain carries multiple acu1 copies. (TIF) [file ppat.1004272.s008.tif]

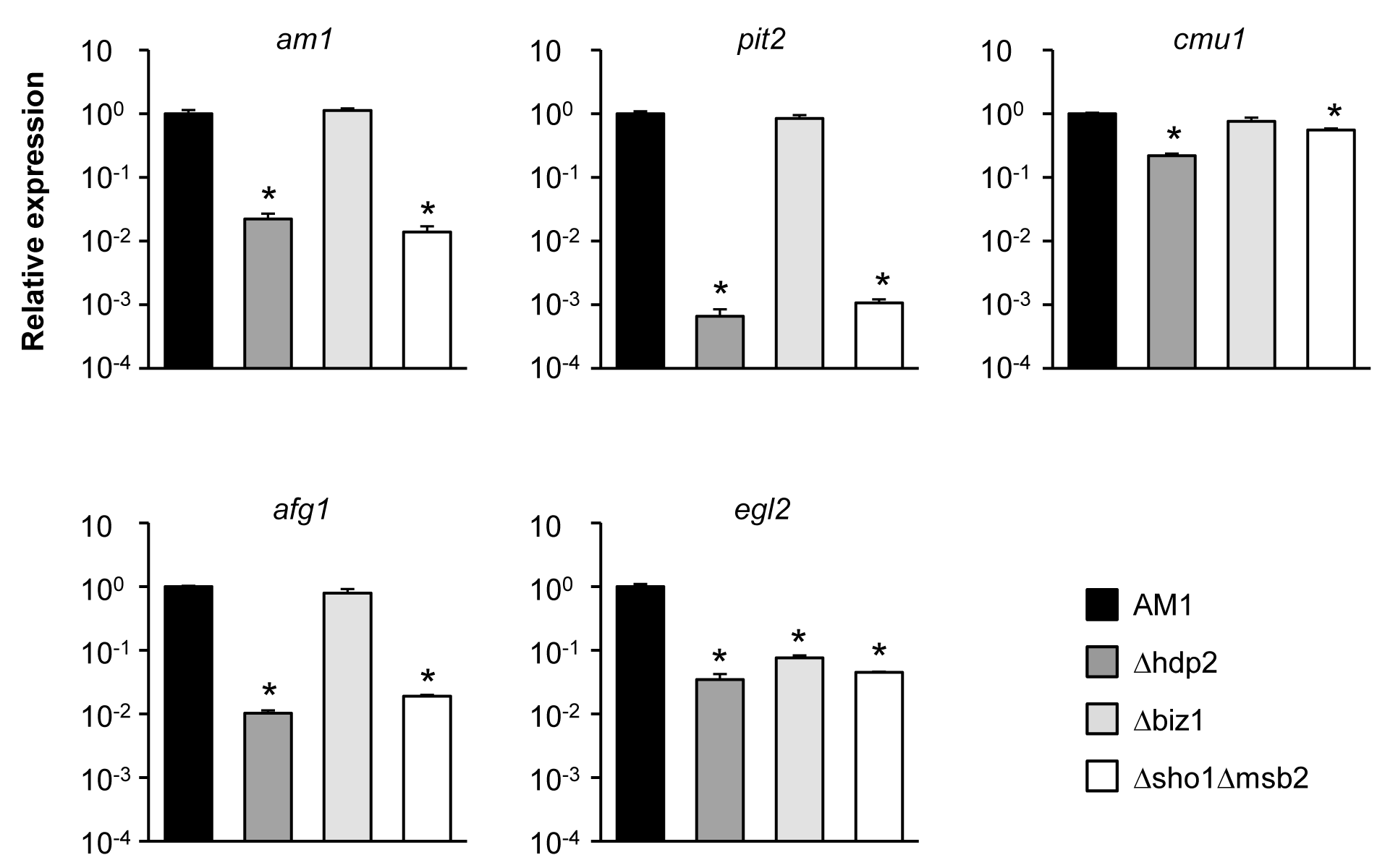

Supplement: Figure S9 — Expression of genes encoding secreted proteins in hdp2 and biz1 mutants. The indicated strains were sprayed on ParafilmM with 100 µM HDA and incubated for 12 h. Relative expression was determined by qPCR for the appressoria marker gene am1, the effector genes pit2 and cmu1, and the CWDE genes afg1 and egl2. For each gene the expression in the AM1 strain was set to 1 and the ratio to the expression in Δhdp2, Δbiz1 and Δsho1Δmsb2 strains calculated. The experiment was conducted in three replicates and the same scale is used for all genes. Error bars denote standard error. *Significant difference (p<0.05, student's t). (TIF) [file ppat.1004272.s009.tif]
