# Supplementary material for: Plant Surface Cues Prime Ustilago maydis for Biotrophic Development
Source: PLoS Pathog. 2014 Jul 17;10(7):e1004272. doi: 10.1371/journal.ppat.1004272 (PMC4102580; doi:10.1371/journal.ppat.1004272)
Supplement: Table S8 — U. maydis strains used in this study. (DOCX) [file ppat.1004272.s017.docx]

**Table S8. *U. maydis* strains used in this study**

| **Strain** | **Genotype** | **Resistence^a^** | **Reference** |
| --- | --- | --- | --- |
| SG200 | *a1:mfa2 bW2bE1* | P | [21] |
| AM1 | *a1:mfa2 bW2bE1 ip^R^[P_um01779_:gfp(3x)]ip^S^* | P, C | [34] |
| AM1Δsho1Δmsb2 | *a1:mfa2 bW2bE1 ip^R^[P_um01779_:gfp(3x)]ip^S^* Δ*sho1* Δ*msb2* | P, C, H, N | [36] |
| AN1 | *a1:mfa2 bW2bE1 P_um01779_:gfp(3x)* | P, N | This study |
| AN1ΔgatA | *a1:mfa2 bW2bE1 P_um01779_:gfp(3x)* Δ*gatA* | P, G | This study |
| AN1Δgad1 | *a1:mfa2 bW2bE1 P_um01779_:gfp(3x)* Δ*gad1* | P, G | This study |
| AN1Δacu1 | *a1:mfa2 bW2bE1 P_um01779_:gfp(3x)* Δ*acu1* | P, N, G | This study |
| AN1Δacu2 | *a1:mfa2 bW2bE1 P_um01779_:gfp(3x)* Δ*acu2* | P, N, H | This study |
| AN1Δacu1Δacu2 | *a1:mfa2 bW2bE1 P_um01779_:gfp(3x)* Δ*acu1* Δ*acu2* | P, N, G, H | This study |
| AN1Δacu1/acu1 | *a1:mfa2 bW2bE1 P_um01779_:gfp(3x)* Δ*acu1 ip^R^[P_acu1_:acu1]ip^S^* | P, N, G, C | This study |
| AN1Δacu2/acu2 | *a1:mfa2 bW2bE1 P_um01779_:gfp(3x)* Δ*acu1 ip^R^[P_acu2_:acu2]ip^S^* | P, N, H, C | This study |
| AN1Δukc2 | *a1:mfa2 bW2bE1 P_um01779_:gfp(3x)* Δ*ukc2* | P, N, H | This study |
| AN1Δukc2/ukc2 | *a1:mfa2 bW2bE1 P_um01779_:gfp(3x)* Δ*ukc2 ip^R^[P_ukc2_:ukc2]ip^S^* | P, N, H, C | This study |
| AN1Δhdp2 | *a1:mfa2 bW2bE1 P_um01779_:gfp(3x)* Δ*hdp2* | P, N, H | This study |
| AN1Δhdp2/hdp2 | *a1:mfa2 bW2bE1 P_um01779_:gfp(3x)* Δ*hdp2 ip^R^[P_hdp2_:hdp2]ip^S^* | P, N, H, C | This study |
| AN1Δaiz1 | *a1:mfa2 bW2bE1 P_um01779_:gfp(3x)* Δ*aiz1* | P, N, H | This study |
| AN1Δaiz2 | *a1:mfa2 bW2bE1 P_um01779_:gfp(3x)* Δ*aiz2* | P, N, H | This study |
| AN1Δaiz3 | *a1:mfa2 bW2bE1 P_um01779_:gfp(3x)* Δ*aiz3* | P, N, H | This study |
| SG200Δafg1 | *a1:mfa2 bW2bE1* Δ*afg1* | P, G | This study |
| SG200Δafg2 | *a1:mfa2 bW2bE1* Δ*afg2* | P, H | This study |
| SG200Δafg3 | *a1:mfa2 bW2bE1* Δ*afg3* | P, G | This study |
| SG200Δ3afg | *a1:mfa2 bW2bE1* Δ*afg1* Δ*afg2* Δ*afg3* | P, N, H, G | This study |
| SG200Δ3afg/afg1 | *a1:mfa2 bW2bE1* Δ*afg1* Δ*afg2* Δ*afg3 ip^R^[P_afg1_:afg1]ip^S^* | P, N, H, G, C | This study |
| SG200Δ3afg/afg2 | *a1:mfa2 bW2bE1* Δ*afg1* Δ*afg2* Δ*afg3 ip^R^[P_afg2_:afg2]ip^S^* | P, N, H, G, C | This study |
| SG200Δ3afg/afg3 | *a1:mfa2 bW2bE1* Δ*afg1* Δ*afg2* Δ*afg3 ip^R^[P_afg3_:afg3]ip^S^* | P, N, H, G, C | This study |
| AM1Δ3afg | *a1:mfa2 bW2bE1 ip^R^[P_um01779_:gfp(3x)]ip^S^* Δ*afg1* Δ*afg2* Δ*afg3* | P, C, N, H, G | This study |
| SG200Δ3egl | *a1:mfa2 bW2bE1* Δ*egl1* Δ*egl2* Δ*egl3* | P, G, H, N | This study |
| AM1Δ3egl | *a1:mfa2 bW2bE1 ip^R^[P_um01779_:gfp(3x)]ip^S^* Δ*egl1* Δ*egl2* Δ*egl3* | P, C, G, H, N | This study |
| ^a^ Phleomycin (P), Hygromycin (H), Genticin (G), Nourseothricin (N), Carboxin (C) | | | |
